# Museomics of an extinct European flat oyster population

**DOI:** 10.1038/s41598-025-96743-8

**Published:** 2025-04-22

**Authors:** Christine Ewers, Dirk Brandis, Nicolas da Silva, Sarah Hayer, Alex Immel, Zoe Moesges, Julian Susat, Montserrat Torres-Oliva, Ben Krause-Kyora

**Affiliations:** 1 Zoological Museum, Kiel University, Hegewischstraße 3, 24105 Kiel, Germany; 2 Institute of Clinical Molecular Biology, Kiel University, Rosalind-Franklin-Straße 12, 24105 Kiel, Germany

**Keywords:** Evolutionary genetics, Next-generation sequencing, Conservation biology, Molecular ecology

## Abstract

Understanding the factors that predispose species and populations to decline and extinction is a major challenge of biodiversity research. In the present study, we investigated the historical population genomics of an extinct population of the European oyster (*Ostrea edulis* L.) from the Wadden Sea collected between 1868 and 1888, and compared it to French and English populations sampled at the same time. Our museomic results indicate that the now-extinct population was genetically isolated from the French and English populations and showed signs of local adaptation in the form of Fst outlier loci between the Wadden Sea and the other two populations. Thus the Wadden Sea oysters may have been predisposed for extinction because they were not naturally replenished from other populations. A comparison of population-wide genomic diversity may hint towards a sudden population contraction of the Wadden Sea population, possibly being the result of stronger - or earlier - population decline in this population than in the others. In summary, our historical population genomic exploration hints at some potential causes of population decline in flat oysters from the Wadden Sea, which might have led to their extinction.

**Supplementary Information:**

The online version contains supplementary material available at 10.1038/s41598-025-96743-8.

## Introduction

Throughout the anthropocene, biodiversity has been lost at a rapid rate, ultimately leading to the extinction of thousands of species^1–3^. Before species go extinct globally, local populations are lost over time^3–5^. Identifying the factors that predispose populations to extinction will benefit conservation biology and aid in the identification of at-risk biodiversity^6^. Main anthropogenic drivers of biodiversity loss are climate change, overexploitation, habitat degradation, increasing disease prevalence and non-native species^3,7,8^. In concert with these external anthropogenic drivers, several intrinsic population-specific factors may expedite local extinction: small population size, low genetic diversity, genetic isolation and high specialization to local conditions in the absence of plasticity^9,10^. Small isolated populations have lower genetic diversity than well-connected meta-populations, and lack an inflow of potentially beneficial variants^11,12^. On the other hand, the inflow of variants can also be detrimental if the number of immigrants is low and happens sporadically, as in the wolves on Isle Royale^12^. Moreover, increased gene flow can swamp out locally adapted alleles^13,14^. Low genetic diversity may limit the potential for the timely adaptation to rapid environmental changes^10,15,16^. Small population size may increase inbreeding depression and genetic load, as shown for the wooly mammoth population on Wrangel Island^12,16,17^. Genetic load describes the decrease of population fitness due to the rise of deleterious alleles, which determines the severity of inbreeding depression and extinction risk^16–18^. Alternatively, deleterious alleles may be purged efficiently in slowly declining or small populations, leading to a reduction of genetic load and inbreeding depression^19,20^. The same factors may prevent recolonization because propagules are not dispersed to isolated habitats, and/or do not have the genetic adaptations required for successful recolonization^9^.

Population genomic comparisons of extinct and extant populations can identify intrinsic drivers of extinction without the confounding effects of evolutionary history that hamper interspecies comparisons. Natural history collections represent one of the few resources to investigate extinct and historical biodiversity; they house historical specimens of many extinct and endangered species^21,22^. Recent advances in ancient and historical DNA sequencing and data analysis make it now feasible to actually attempt such comparisons - but the few existing studies do not confirm the expected population genetic patterns, particularly of low genetic diversity and population isolation in now-extinct populations. Two extinct Australian populations of the New Holland mouse had genomic diversity comparable to contemporary extant populations, though overall population connectivity is limited^23^. Similarly, a microsatellite analysis of herbarium specimens revealed similar genetic diversity in an extinct and still extant mustard populations that were well-connected by gene flow, though the extinct population declined in diversity prior to extinction^24^.

Especially the decline of keystone species and ecosystem engineers has large ecological and economic impacts, altering ecosystem functions and services substantially^25^. One such group of declining ecosystem engineers are oysters^26^. Like many other oyster species, the European oyster *Ostrea edulis*L. has decreased in abundance throughout much of its range in the past 150 years^27–29^. Most drastically, it went locally extinct in the German and Danish Wadden Sea around 1930, presumably due to a combination of overfishing, harsh winters and disease^29,30^. It has not yet recolonized this habitat, even though harvesting of oysters ceased decades ago, harsh winters have become an exception and much of the Wadden Sea is now a protected nature reserve. Competition for food or space between the non-native Pacific oyster *Magallana gigas *(Thunberg, 1793) and the native European oyster is unlikely to have impacted the re-establishment of the European oyster: the Pacific oyster is a primarily intertidal species, while the European oyster occurred subtidally in the Wadden Sea^31,32^. Similarly, the non-native slipper limpet *Crepidula fornicata *(L., 1758) did not cause the local extinction of the European oyster in the Wadden Sea directly, as the oyster began to decline prior to arrival of the slipper limpet^33^.

**Fig. 1:**
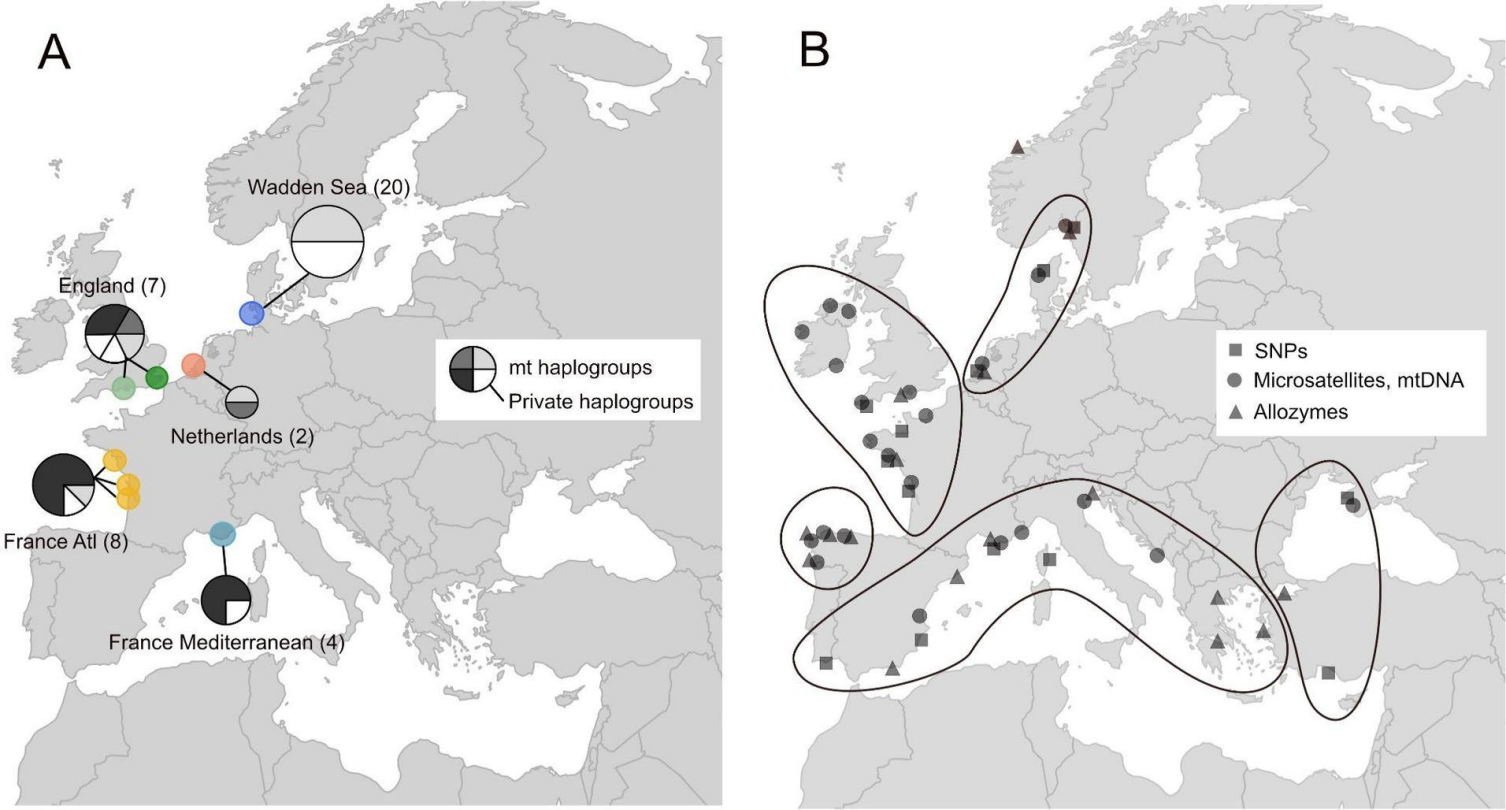
Historical and present-day genetic population structure of the European oyster *Ostrea edulis*. **A**: historical distribution of mitochondrial genome haplogroups at the end of the 19 th century highlighting the uniqueness of the Wadden Sea population^30^. **B**: present-day genetic population structure synthesized from several studies highlighting the presence of at least five distinct genetic clusters^28,35,37–39^. Outlines denote clusters of genetically similar oyster populations. The map was generated with the function ‘map’ of the package ‘maps’ in the R environment^40^.

Genetic isolation of the Wadden Sea population could have played a role in its demise. Analyses of mitochondrial genomes extracted from 150-year-old European oyster shells, including shells from the now-extinct Wadden Sea population, indicated limited connectivity between the Wadden Sea population and the French-English populations (Fig. 1A): while English and French oysters were not differentiated from each other, Wadden Sea oysters had a distinct genetic makeup with one private haplogroup^34^. Extant oyster populations are split into five geographic groups (Fig. 1B): a Scandinavian-Dutch group in the North Sea, a French-English group, a northern Iberian group, a southern Iberian-Mediterranean group and a Black Sea group^28,35–39^. The now-extinct Wadden Sea population was in the geographic center of the North Sea group. The five groups are delimited by oceanographic fronts and biogeographic barriers, which likely limit larval dispersal^38^. At the same time, this does not preclude the possibility of local adaptation at certain loci. One example indicating local adaptation is the allozyme locus arginine kinase, which has a strong latitudinal cline throughout the range of European oysters^38,39^. Such correlations between latitude and functionally relevant genetic variation can indicate adaptation to environmental variables that co-vary with latitude. Another example identified several SNPs that had similar allele frequencies in the North Sea and the Black Sea, but that differed strongly from the allele frequencies observed in the Atlantic and Eastern Mediterranean^37^. Two hypotheses may explain this pattern: ancient gene flow between the Black Sea and the North Sea populations, and/or convergent adaptation at these or linked loci.

The aim of the present study was to understand how the genetic diversity, the potential for local adaptation and patterns of geographic isolation of the Wadden Sea population might have played a role in its extinction in the context of historical populations that survived until today, namely populations from France, England, and the Netherlands. The French Atlantic population we were able to include into the analysis is severely depleted today^21^. English populations from the English Channel and Thames have been large enough for an ongoing small fishery, though some have declined rapidly in the last years^22^. In the Netherlands, oysters have persisted in enclosed estuaries such as the Oosterschelde, from where the oysters from our analyses originated, until today^41^. These populations were sampled between 1868 and 1888 by the German zoologist Karl-August Möbius. The original aim of this collection was to understand oyster ecology and the conditions needed for successful oyster culture. The collections survived until today and are housed at the Zoological Museum in Kiel, Germany^33,42^. Today, this collection represents a very unique resource to study declining and extinct populations, unmatched in its geographic extent, completeness of documentation and age. The dawn of museomics and bioinformatic approaches suited for low coverage whole genome sequences (lcWGS) make it now possible to extract genome-scale data from these invaluable specimens and analyze their genomic structure and adaptive signatures^34,43–50^.

## Results

### Genome assembly and annotation

Since the beginning of our investigations, three chromosome level genome assemblies for the European oyster have become available^51–53^. A preliminary analysis based on mappings against our draft genome indicated that the fragmentation of our genome and potentially its geographic origin biased our results (Supplementary Information S1). Therefore we mapped all reads against the chromosome-level assembly of Gundappa et al.^52^. We still report the results of our draft genome assembly here as it may be of use for comparative studies. The Illumina run of the Genomics Chromium library generated a total of 623 million barcoded reads. The final *O. edulis *draft assembly of an oyster collected in the Danish Limfjord has a size 967 Mbp, with 14,796 scaffolds larger than 10 Kbp and a scaffold N50 of 77.76 Kbp. The final annotation comprises a total of 16,615 gene models and 45,064 predicted transcripts. A search using BUSCO v5^54^ (metazoan_odb10) indicated that this gene set contains 91.2% single-copy universal metazoan genes (78.5% complete and 12.7% fragmented).

### Whole genome shotgun sequencing, quality control and mapping

**Fig. 2:**
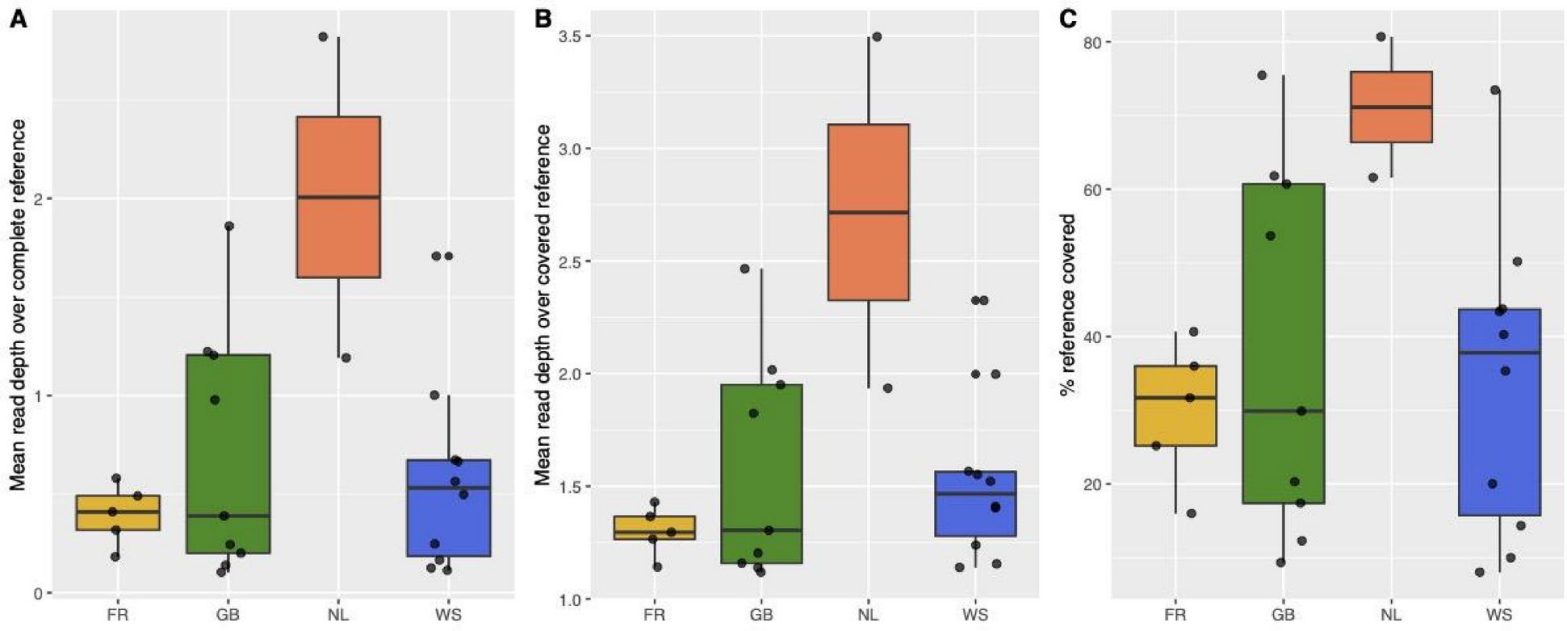
Mapping results. **A**: Mean read depth over the complete reference genome. **B**: mean read depth over the covered reference genome. **C**: % of the reference genome covered by at least 1 read.

The sequencing efforts for each of the 26 oysters collected between 1868 and 1888 in the Danish-German Wadden Sea, England and France generated 6,289,842 to 75,541,394 reads per run (Supplementary Tables S1 and S2 for sampling information). Between 9.357% and 83.352% (median = 36.488%) of the reads mapped to the nuclear genome of *O. edulis*^52^. After combining the runs for each sample, median genome coverage was 0.4478x, and ranged from 0.1008 to 2.8314x (Supplementary Table S3). Coverage was comparable across populations; only the two Dutch oysters had a significantly higher genome coverage than the other populations (Fig. 2A, B), possibly because these samples were less degraded than the others. None of the oysters showed substantial DNA damage (see Supplementary Figs. S1 - 4 for examples). On average, 36% of the reference genome was covered by at least one read (Fig. 2C). A total of 243,005 polymorphic sites passed our genotype filters, i.e. had SNP p-value < 1e- 6, mapping quality > 30, minQ > 25, minor allele frequency > 0.05 and were present in 20 or more individuals. The SNP p-value is based on a likelihood ratio test of the site being variable using the population allele frequency^47^. Given the low coverage of our data we based the subsequent analyses on genotype likelihoods instead of hard-called genotypes. The historical mitochondrial genomes had a median coverage of 24.24x (range: 0.86–549.38), and 19 samples were covered with at least 4x across more than 90% of the genome.

### Population genetic structure and diversity

**Fig. 3:**
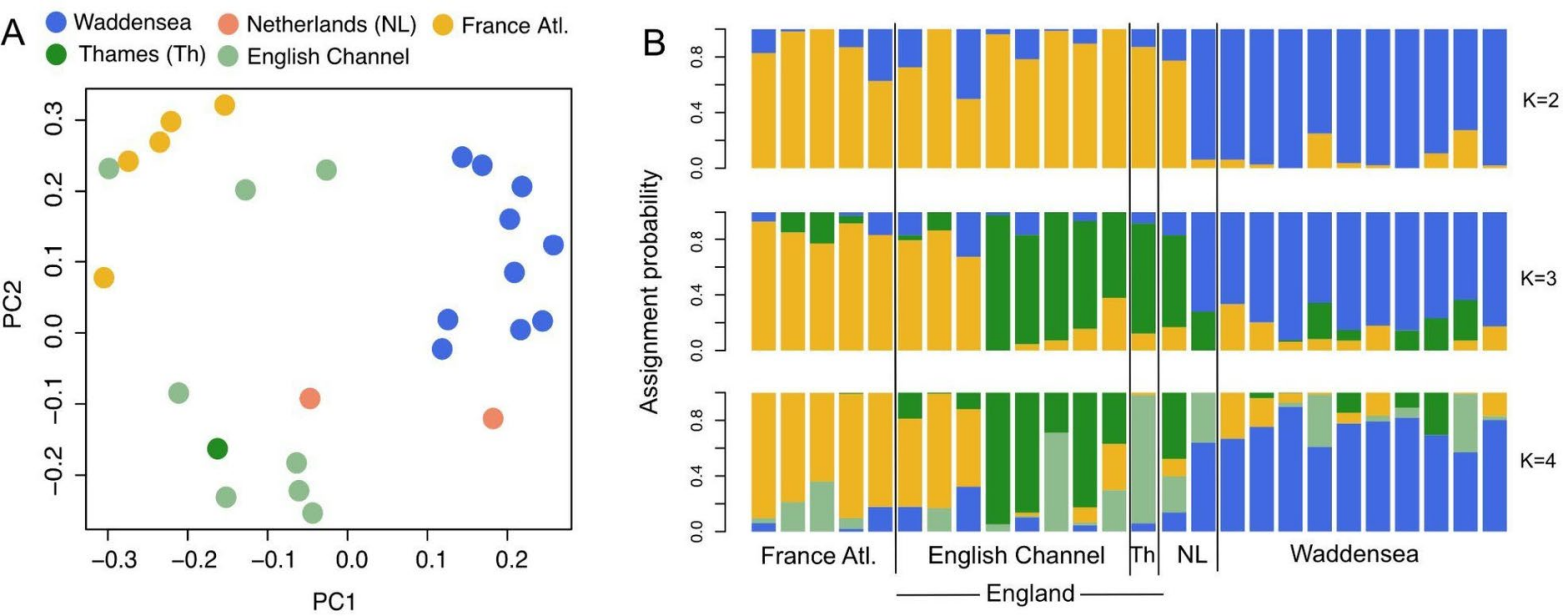
Nuclear population genomic structure of European oysters collected at the end of the 19 th century. **A**: Principal component analysis. Samples are colored based on their sampling locality. In subsequent analysis, Thames and English Channel samples are analyzed as a single “English” population. **B**: Admixture plot. Each bar is one individual, and the colors denote the assignment probability to K ancestral populations.

Omitting samples with ultra-low coverage (< 0.5x average genome coverage) did not change results of the principal component analysis (PCA) qualitatively (Supplementary Fig. S5 A), and we thus present the results for all samples with a nuclear genome coverage above 0.1 (*n* = 26). The first principal component of the PCA separated the now-extinct Wadden Sea oysters from the French and English oysters. The second principal component separated the French and English oysters except for three English oysters collected in the English Channel that clustered with the French Atlantic oysters (Fig. 3A). The two Dutch oysters clustered with the Wadden Sea and English populations, respectively (Fig. 3A).

For the admixture analysis, K = 3 had the highest delta K value (1135.6372), followed by K = 5 (927.8341), K = 2 (912.6502) and K = 4 (895.1018). The results of the admixture analysis were concordant with the PCA results: the Wadden Sea oysters and one of the Dutch oysters were assigned predominantly to a single genetic cluster, most of the English oysters and one of the Dutch oysters to a second cluster, and the French and three English oysters to a third genetic cluster without much admixture in these three English oysters (Fig. 3B). The probability of French and British oysters being actually admixed but the samples being coincidentally collected from the two clusters is only 0.0005. Omitting the three British oysters from the French cluster, the genome-wide unweighted Fst was 0.108631 between the Wadden Sea and English populations (WS-GB), 0.1119 between the Wadden Sea and French populations (WS-FR) and 0.0566 between the French and English populations (FR-GB).

We tested for HWE and inbreeding in the Wadden Sea, French and English populations separately. Of 29,166 high-quality SNPs shared between all three populations, 2% (583 loci) showed significant heterozygote excess in the French population, whereas 8% (2,341 loci) did in each of the English and Wadden Sea populations. The French population had no SNPs experiencing significant inbreeding, whereas 0.2% (54 loci) of the SNPs in both the English and Wadden Sea populations showed signs of inbreeding (excess of homozygotes). The overall proportion of heterozygotes was around 40% in all three populations. Given the small sample size (*n* = 5) and overall lower genome coverage of the French oysters (mean coverage across the whole genome = 0.4), the lower number of significant values in the French population is possibly the result of low statistical power.

**Fig. 4:**
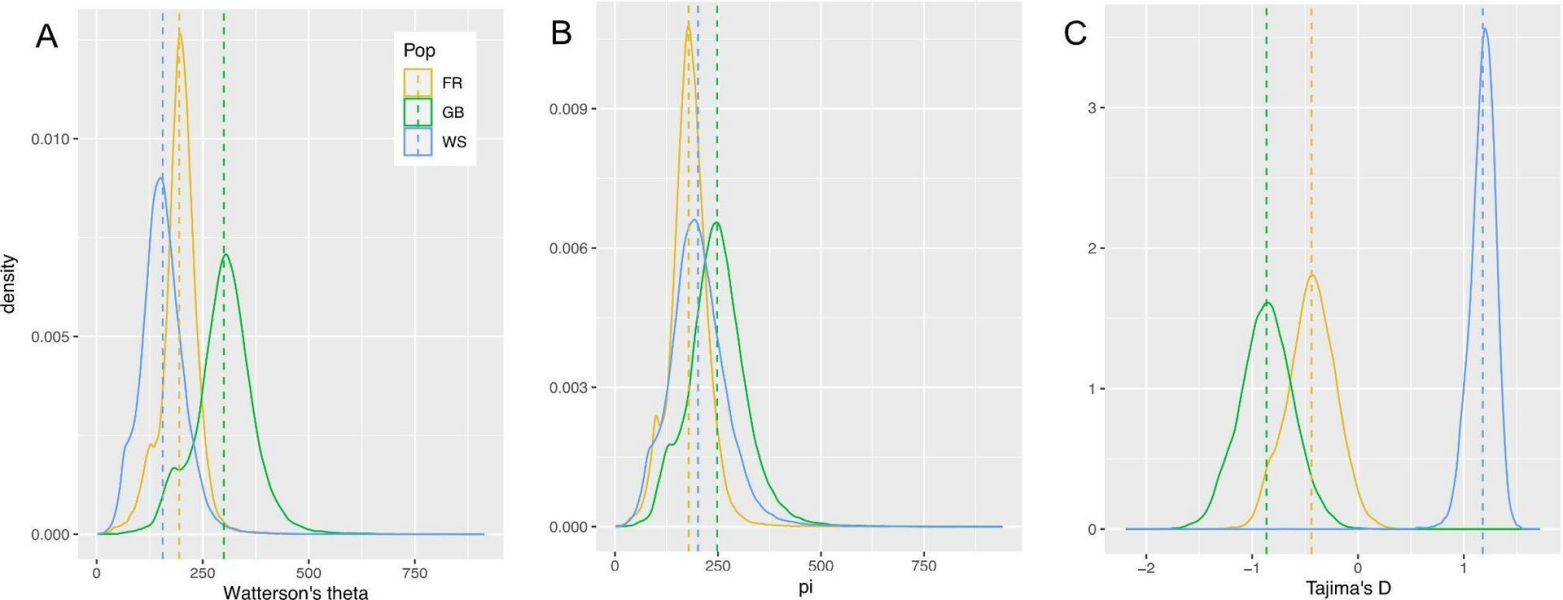
Genome-wide genetic diversity. Density distributions of sliding window estimates. **A**: Watterson’s theta, **B**: π, **C**: Tajima’s D. Dashed lines show the mean values. Abbreviations: FR = France, GB = England, WS = Wadden Sea.

For all sliding window scans of genomic diversity, we analyzed the Wadden Sea, French and English populations separately. We did not analyze the Dutch oysters because of their low sample size (*n* = 2) and the clustering of the Dutch oysters with the English and Wadden Sea populations. Furthermore, we excluded the three English oysters that clustered with the French oysters from the analysis. We estimated two different measures of genomic diversity for 89,276 sliding windows: Watterson’s theta estimates the number of polymorphic (or segregating) sites, while π estimates nucleotide diversity, i.e. the average number of pairwise differences between all pairs of individuals. The Wadden Sea population had on average slightly fewer polymorphic sites than the other two populations (Fig. 4A) but the number of pairwise differences was similar for all populations (Fig. 4B). This means that rare alleles were scarce in the Wadden Sea population, resulting in a mean positive Tajima’s D of 1.178, whereas the French and English populations had slightly negative Tajima’s D values of − 0.439 and − 0.864, respectively (Fig. 4C).

**Fig. 5:**
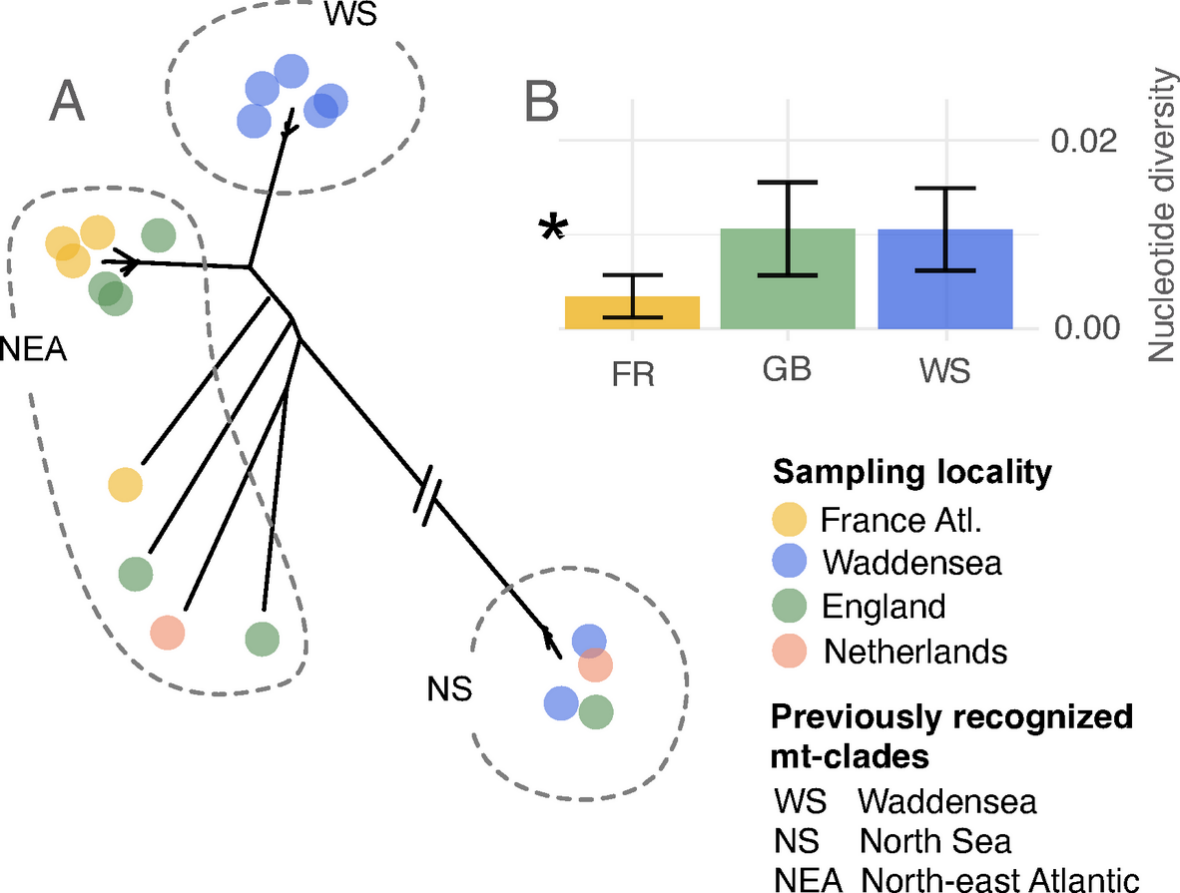
Mitochondrial population genetic diversity of historical European oysters. **A**: phylogenetic reconstruction (maximum likelihood tree) showing at least three distinct genetic clusters. Branch to the NS clade was shortened to facilitate visualization. Clade names are taken from Hayer et al.^34^ **B**: mitochondrial nucleotide diversity per sampling locality indicating low mitochondrial genetic diversity in the French Atlantic population. Error bars denote confidence intervals. Asterisk indicates significantly lower nucleotide diversity in the French population.

The phylogenetic tree reconstructed for the 19 mitochondrial genomes that passed our quality checks was congruent with the reconstruction of Hayer et al.^34^. Most of the mitochondrial genomes fell into one of three distinct clades: one clade contained only Wadden Sea oysters (the “WS” clade of Hayer et al. 2021), a second clade French and English oysters (“NEA” clade), and the third clade contained English, Wadden Sea and Dutch oysters (“NS” clade) (Fig. 5A). The mitochondrial nucleotide diversity of the Wadden Sea population (0.010500, *n* = 7) was not significantly different from the English population (0.010613, *n* = 6), indicated by overlapping confidence intervals (Fig. 5B). The French population had a significantly lower nucleotide diversity than the two other populations (0.003459, *n* = 4), as it did not contain any haplotypes of the most divergent NS clade (Fig. 5A).

### Genomic signatures of selection

**Fig. 6:**
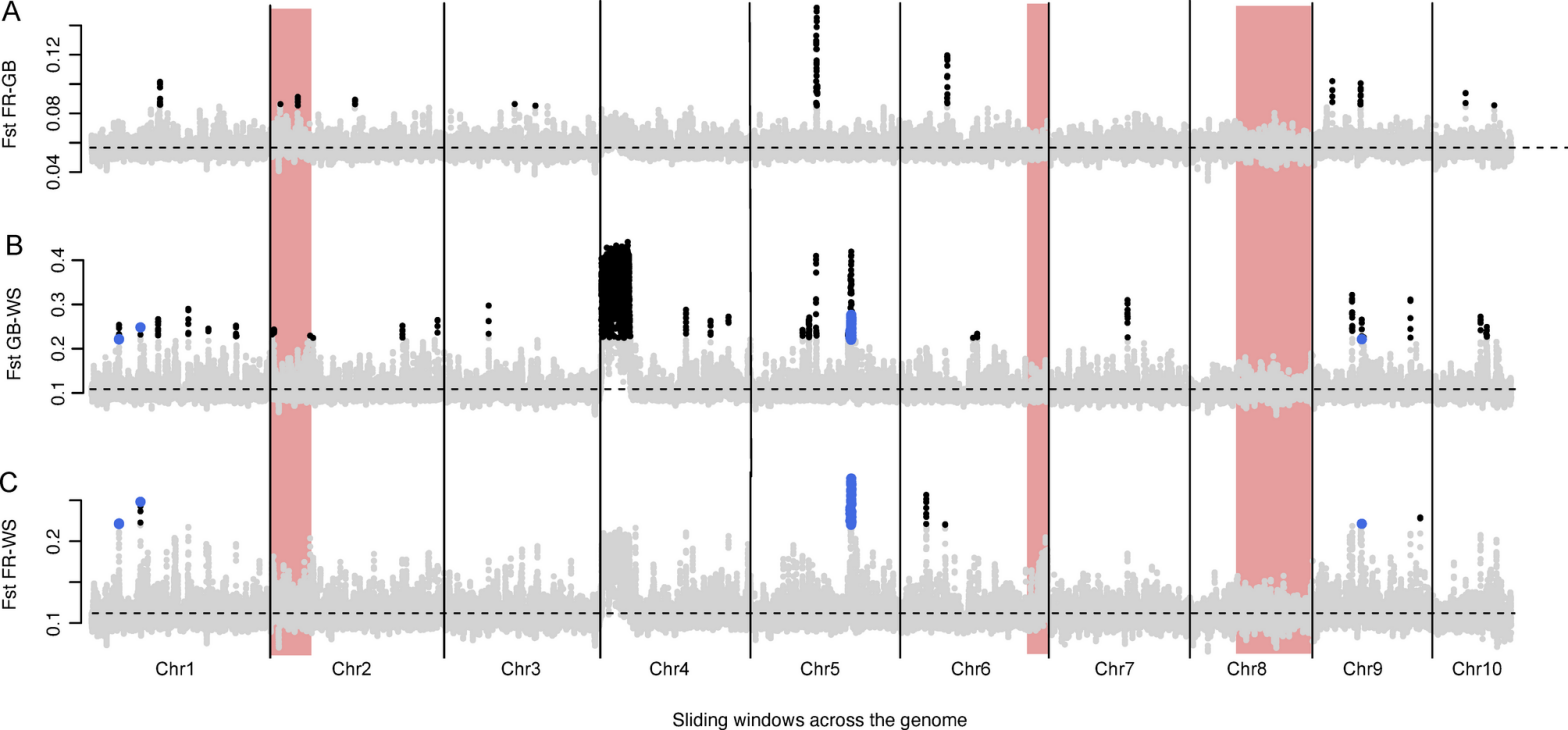
Fst sliding window genome scan to identify potential regions of local adaptation. Red bars denote large chromosomal inversions that can lead to increased Fst between populations. **A**: Fst between French Atlantic and English oysters. **B**: Fst between English and Wadden Sea oysters. **C**: Fst between French Atlantic and Wadden Sea oysters. Blue dots represent sliding window estimates that were outliers in both comparisons with the Wadden Sea population. Black dots represent significant outliers in one of the interpopulation comparisons.

Fst between all three population pairs was estimated for 89,299 sliding windows. Of these, 52 were significant outliers based on Rosner’s generalized extreme Studentized deviate test after Bonferroni correction for WS-FR, 1844 for WS-GB and 88 for FR-GB (Fig. 6). The Manhattan plots show that this is a conservative estimate for WS-FR; in particular a large region at the beginning of chromosome four had high Fst values but was not identified as an outlier region. Thirty-five of the outliers were shared between WS-FR and WS-GB and located on seven regions of the genome (Fig. 6B, C), but none of these genomic regions were identified as outliers for FR-GB. A PCA based on the outlier regions identified in both comparisons with the Wadden Sea population recapitulated the pattern identified for the complete genomes (Fig. 3A) but clustering was less pronounced, possibly due to the smaller number of SNPs (Supplementary Fig. S5B). The population genetic structure was also recapitulated when excluding outlier regions in the PCA (Supplementary Fig. S5 C).

Three large inversions have been identified in the *O. edulis *genome^37^ that could cause Fst outliers as well. In the assembly of Boutet et al.^53^, they are located at the end of chromosomes 3 and 8, and the beginning of chromosome 5. This translates to the end of chromosomes 6 and 8 and the beginning of chromosome 2, respectively, in the assembly of Gundappa et al.^52^ that we mapped against. None of our outlier regions fell within these inversions (Fig. 6).

PCAdapt did not identify any SNPs under selection after Bonferroni correction, neither for the first nor second principal component axes (Supplementary Fig. S6). The highest observed selection coefficient (D) was 18. The smallest Bonferroni corrected p-value was 1.802 × 10^−5^, while significant values would have had to be smaller than 10^−6^. This may be the result of the relatively small sample size and/or the use of individual SNPs instead of sliding windows as in the Fst outlier test.

**Fig. 7:**
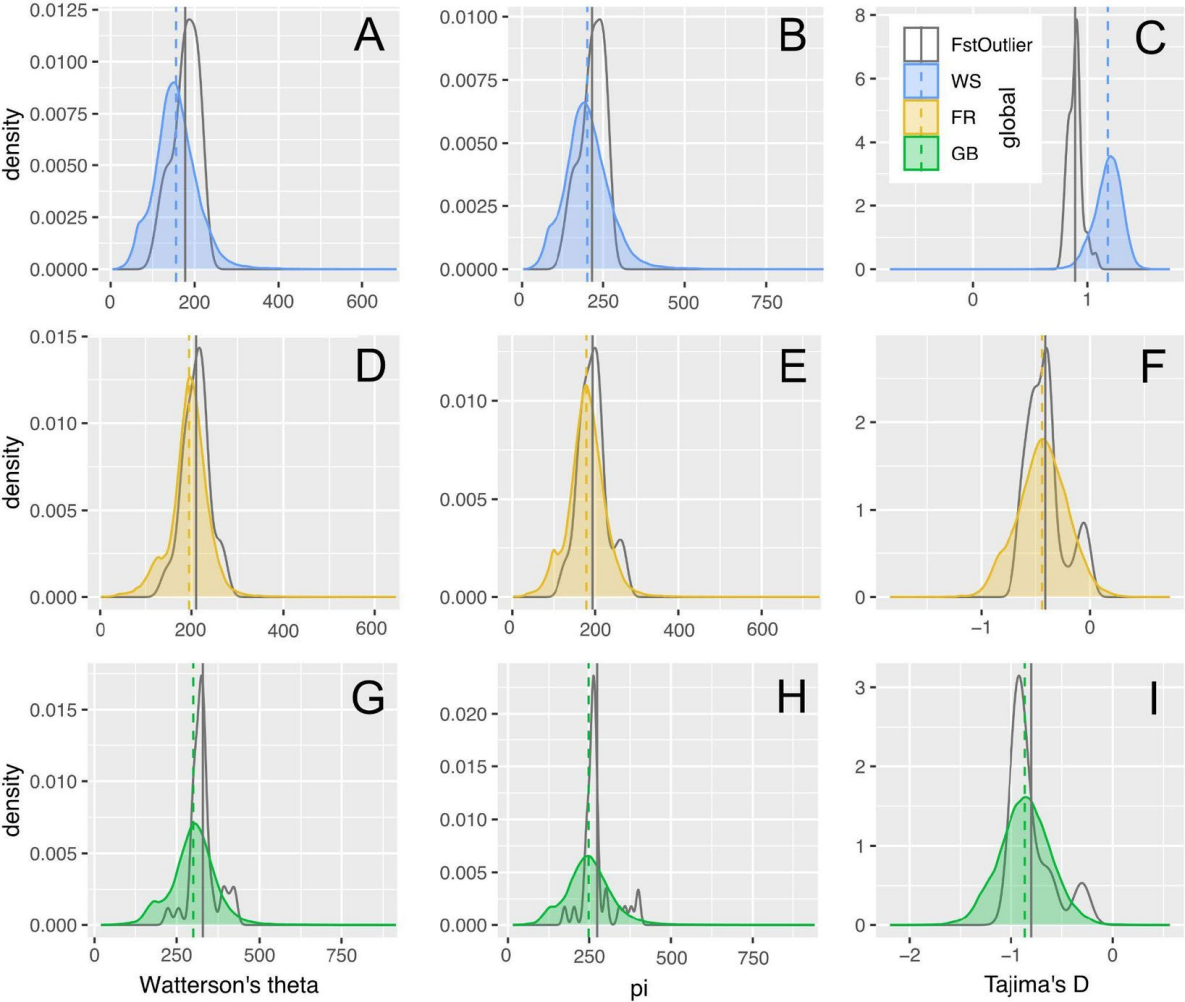
Density distribution of diversity metrics for the Fst outlier regions of the Wadden Sea population (gray line) in comparison to the genome-wide sliding window estimates (in color). Vertical lines represent the mean values. Watterson’s theta: **A**, **D**, **G**; Nucleotide diversity π: **B**, **E**, **H**; Tajima’s D: **C**, **F**, **I**. Wadden Sea: **A**, **B**, **C**; France: **D**, **E**, **F**; England: **G**, **H**, **I**.

The expectation that Fst outlier regions have negative Tajima’s D values was not reflected in our data (Fig. 7C, F, I). However, the Wadden Sea outlier regions did have on average smaller Tajima’s D values than the rest of the genome. This was not caused by overall lower diversity (Fig. 7A, B) in these regions, as expected for selective sweeps. Instead, these regions appear to behave more neutrally in having more similar values of Watterson’s theta and π. The two other populations do not show deviations from the overall genomic pattern in the Fst outlier regions identified for the Wadden Sea population (Fig. 7D-I).

A total of 23 genes and two long-noncoding RNAs (lncRNAs) were located at least partially within the seven consecutive Fst outlier regions identified between the Wadden Sea population and both other populations. Two of these outlier regions were on chromosome one, four regions on chromosome five and one region on chromosome nine (Fig. 6). The regions were between 50,000 and 180,000 bp long and spanned a total of 630,000 bp. While 5 genes and the two lncRNAs had uncharacterized functions, 18 genes had inferred functions. Eight genes were assigned to transmembrane and intracellular transport of ions and nutrients (Table S4), and eleven genes have been implicated to respond to ecological stress in oysters (Table 1, Table S4)^59–64^.

**Table 1:** Genes under putative local adaptation in the Wadden sea population, for which ecological functions have previously been identified for oysters.

Gene	Gene ID	Ref.
Osmoregulation and salinity stress		
Sodium-dependent phosphate transport protein 2B-like	LOC125648921	^61^
Sodium-dependent phosphate transport protein 2B-like	LOC125648922	^61^
Sodium-dependent phosphate transport protein 2B-like	LOC125648923	^61^
Calcium permeable stress-gated cation channel 1-like	LOC125651075	^63^
Neuronal acetylcholine receptor subunit alpha- 9-like	LOC130054664	^64^
Cell apoptosis		
Proteasome subunit alpha type- 3-like	LOC125652196	^63^
Immune response		
Probable G-protein coupled receptor 139	LOC125652017	^62^
Menin-like	LOC125651755	^59^
Stress response		
Seipin-like	LOC125651753	^63^
Putative lipid scramblase CLPTM1	LOC125651750	^63^
2-aminoethylphosphonate–pyruvate transaminase-like	LOC125651440	^63^

Ref. = reference.

## Discussion

The evolutionary history and demography of extinct and threatened biodiversity can elucidate processes and mechanisms that drive species decline, and inform us about the most vulnerable parts of biodiversity. We investigated the population genomic and evolutionary makeup of an extinct oyster population in the Wadden Sea, and compared it to historical populations sampled at the same time in France, England and the Netherlands, where oysters have persisted in small numbers until today.

The historical nuclear genomes clustered mostly by country of origin, indicative of limited gene flow between them. Contemporary population genetic studies based on 16 microsatellite or 203 SNP markers did not identify any differentiation between French and English populations (Fig. 1B)^20,26,28–30^. If this difference is not driven by the larger number of markers used in our study, it suggests an erosion of population genetic structure between France and England in the last 150 years, most likely caused by human actions rather than natural dispersal. On the one hand, translocations between France and England have been carried out frequently from the 19 th century on^52^. They could have altered the genomic makeup of the oysters over the past 150 years. Three English oysters with a French genomic background may substantiate the translocation hypothesis. The high assignment probabilities of these English oysters to the French population suggest that the three individuals were translocated very recently before collection. Alternatively, oyster larvae can disperse naturally from France to England. Though in this case, we would have expected more admixture in the English population as this natural phenomenon would have occurred over long time scales, and caused admixture between English and French oyster populations. Future studies should investigate contemporary divergence with a whole genome approach, and compare it directly to historical levels of divergence based on the same type of data.

The Wadden Sea population was more divergent from the English and French populations than those populations from one another. It was likely isolated from England and France due to limited hydrographic connectivity^65^, concordant with results for historical mitochondrial genomes of the European oyster^34^, and other marine invertebrate species^55–58,66^. In addition, translocations of oysters into the Wadden Sea have not been reported, which could have retained the natural isolation in comparison to the French and English populations^27,67–69^. The fact that one of the Dutch oysters fell into the Wadden Sea genomic cluster may indicate immigration of Danish-German Wadden Sea oysters to the Netherlands, which is likely given their geographic proximity. The Dutch oyster that fell into the English cluster, on the other hand, could have originated from the massive restocking efforts with English oysters or from natural dispersal^67,70^. As argued earlier, ongoing natural dispersal should have caused admixture of the Dutch oysters. Both oysters we were able to include into our analyses were stocked oysters from the Oosterschelde according to Möbius’ records, but whether these oysters were imported from different regions is not known.

We identified a relative lack of rare alleles in the Wadden Sea population, and positive Tajima’s D values across the genome. These results should be considered with caution because of the low genome coverage and small sample sizes. However, genome coverage was equally low across all investigated populations. Biologically relevant reasons for this pattern are sudden population contraction, balancing selection or the inclusion of genetically divergent samples^71,72^. Our analyses did not indicate any cryptic population genetic structure within the Wadden Sea population, eliminating the last hypothesis. Balancing selection affects only a small fraction of the genome^73^and does not lead to a genome-wide pattern of positive Tajima’s D values, making the second hypothesis unlikely. Moreover, we did not detect higher levels of heterozygote excess in the Wadden Sea population in comparison to the English population. This leaves sudden population contraction as the most likely reason for the observed population genetic pattern. This could denote the demographic beginning of the extinction of the Wadden Sea oyster, which may have been triggered by overfishing and recurring reproductive failure^28,29,42^.

To understand the contribution of adaptive processes to the differentiation of the historical populations and the Wadden Sea population in particular, we conducted genome scans for signatures of selection. We found that parts of the genome have undergone more rapid divergence (higher Fst values) than the remainder of the genome, which may suggest local adaptation. However, there are a number of caveats that need to be discussed. Confidently revealing local adaptation specific to the Wadden Sea would have ideally been based also on comparisons between this population and other North Sea populations, i.e. the Scandinavian populations. Unfortunately, several attempts to extract DNA from historical oyster shells from Norway, Sweden and Denmark remained unsuccessful. The comparison with the more distant English and French populations may not only reveal signatures of local adaptation, but also neutral demographic stochasticity or genome architectural variation.

Given the geographic location of the Wadden Sea population, it is possible that the now-extinct Wadden Sea oyster population belonged to the same genetic cluster as present-day oysters from Scandinavia and the Netherlands. In the absence of historical samples from these locations, it may not be possible to exclude the possibility that the population genomics patterns we identified are not unique to the extinct Wadden Sea population, but rather typical of the entire North Sea cluster. However, evidence from Lapègue et al.^37 ^appear to discount this hypothesis, as they identified three large chromosomal inversions that differentiated the Scandinavian-Dutch population from most of Europe but were also present in the Black Sea population^37^. They propose two alternative explanations for this genetic parallelism: historical gene flow between the Black Sea and the North Sea, and convergent adaptation in both regions. However, the outlier regions we identified do not fall within the reported chromosomal inversions, and we found no sign of these reported inversions. This could be due to the low coverage of our data, which tends to underestimate genomic differentiation^47^. On the other hand, we identified a large region of high differentiation at the beginning of chromosome four, which could be an inversion specific to the Wadden Sea population. This inversion might not have been identified by Lapegue et al.^37^ because of their limited number of SNPs but could also have gone extinct with the Wadden Sea population. In the latter case, this divergent region would be an indication of the uniqueness of the Wadden Sea population. Future studies should generate higher coverage data of historical and contemporary oysters to understand the geographic extent and fate of these genomic inversions.

No SNPs were identified as under selection by the PCA-based scan for selection, possibly an artifact of low sample size. In addition, the Fst outlier regions did not have lower genetic diversity than the other parts of the genome as expected under classic models for selective sweeps. These models pertain, however, only to a short time window during and after the sweep, and erode quickly in highly recombining regions^74^.

Thus, taken together, our genome scans hint toward the possible presence of local adaptation in the Wadden Sea population and to the fact that it might have resulted from selection that has been ongoing for some time and/or from soft selective sweeps. The historical failure of restocking efforts of the Wadden Sea population with oysters from France, England and the Netherlands in the 1930 s may also point to local adaptation of the Wadden Sea population, and an inability of foreign oysters to survive and reproduce in this region^24^^[,75^. Thus local adaptation may have played a role in differentiating the Wadden Sea population functionally, and limit the success of recolonization efforts. Moreover, much of the Wadden Sea has been a protected area since 1985 with limited disturbance from fishing and other anthropogenic actions.

Going a step further and searching for genes in the Fst outlier regions shed light on the potential targets of local adaptation. The identified genes had diverse functions, with a high proportion of genes involved in transmembrane and intracellular transport. We identified several outlier genes with unknown functions, which is quite common in non-model organisms, as assigning functions requires extensive experimental work. That said, oysters are better investigated than many other non-model organisms due to their economic importance, and in addition to genes with unknown functions, several outlier genes have previously been identified as differentially expressed at the transcriptome level or differentiated at the DNA level between oyster populations exposed to different environments, in particular with regard to salinity^59,61–64^. The hypothesis of salinity adaptation is strengthened by the variability of ocean surface salinity between our investigated populations, and particularly low salinities in the Wadden Sea (https://salinity.oceansciences.org/smap-salinity.htm#, accessed August 14, 2024).

It appears curious that the Wadden Sea has never been recolonized from extant populations in its vicinity. Given the outlier tests, we conclude that adaptations specific to the Wadden Sea population might have existed, preventing oysters from other populations to become established. Alternatively, or simultaneously, the larvae from surrounding populations never reached the Wadden Sea in sufficient quantities. The genetic differentiation of the Wadden Sea oysters is an indication of this and is further corroborated by the currents in the region^75,76^. The overall currents flow northwards from the English Channel via the Wadden Sea to the Skagerrak, making it unlikely that Scandinavian oysters reach the Wadden Sea. The Limfjord, which is the closest still existing population, has only a narrow opening to the North Sea and is situated north of the Wadden Sea^75,76^. Dutch oysters were also not available as larval donors: natural Dutch populations went extinct in the 1950 s, and Dutch oyster fisheries have been sustained in areas that were closed off from the North Sea by means of seed oysters. Only very recently have European oysters been reported from Danish and Dutch offshore wind farms, hinting at a possible re-colonization of the North Sea^77,78^.

## Conclusions

Our analyses shed light on the historical genetic diversity of an extinct population of the European oyster (*O. edulis* L.) from the Wadden Sea, and compared it to historical diversity of French and English oysters. We observe signs of sudden population decline, which could have marked “the beginning of the end” of the Wadden Sea population. Furthermore, the Wadden Sea oysters appear to have been relatively isolated and potentially locally adapted to the unique conditions of the Wadden Sea, possibly its low salinity, though additional populations should be included in future studies. Both factors, isolation and local adaptation, could have accelerated the decline of this population, as foreign propagules may have been unable to reach the region and add to the declining population.

## Methods

### Genome assembly and annotation

At the beginning of this work, a reference genome for the European oyster was not yet available, and our initial aim was the production of a nuclear genome assembly for *O. edulis. *In October 2018, we purchased a fresh individual at Limfjord (Denmark) and extracted 25 mg of soft tissue with the MagAttract HMW DNAKit (Qiagen) following the manufacturer’s protocol. The required fragment length larger than 50 Kbp was measured using Agilent TapeStation 4200 and one 10x Genomics Chromium library was prepared and sequenced on one Illumina HiSeq4000 lane (Illumina, San Diego, CA, USA)^79^. The resulting reads were used to assemble a draft genome using supernova v2.1.1^80^. In order to annotate this draft genome assembly, different evidence datasets were obtained from public repositories and used in a nextflow-based pipeline developed in-house (https://github.com/ikmb/esga*)*^81^. A thorough description of this pipeline can be found on the github page, but in summary, the genome draft was initially repeat-masked using RepeatMasker v2.4.1^82^. Next, annotated proteins of the Pacific oyster *M. gigas*were downloaded from Ensembl (GCA902806645v1)^83^and mapped against the repeat-masked genome using Spaln v2.4.6 and the taxon model “Echinode”^84^. The resulting models were considered high-confidence gene models. In parallel, all reviewed oyster proteins were downloaded from UniProt^85^ and mapped using Spaln, while an *O. edulis*EST dataset (SRR3954443) was downloaded from NCBI and mapped using Minimap2 v2.22^86^. These two sets of alignments were used as evidence-based hints to run Augustus v3.4.0^87^^[,88^. The resulting predicted gene models, together with the previously described high-confidence gene models, were used to finally run EVidenceModeler v1.1.1^89^ and obtain the final set of annotated gene structures.

### Whole genome shotgun sequencing and mapping

DNA extractions in an ultra-clean facility, library preparation and initial shotgun sequencing were carried out by Hayer et al.^34^from historical oyster shells housed at the Zoological Museum in Kiel, Germany. These oysters were sampled alive along European coasts between 1868 and 1888, prior to the local extinction of the Wadden Sea population^42^. Initially, the libraries were shotgun sequenced on 1/50 th of an Illumina HiSeq lane each^34^. We re-sequenced 26 of these libraries with the highest endogenous DNA content on 1/20 th of a lane on the Illumina HiSeq 4000 platform (2 * 75 cycles) to generate higher genome coverage for nuclear genome analysis. The sampling information for all analyzed specimens can be found in Supplementary Tables S1 and 2.

De-multiplexing was performed by sorting reads corresponding to their p7 and p5 combinations using the Bcl2fastq software (Illumina, Inc.). Reads of the initial and the deeper sequencing runs were processed according to published protocols specific for aDNA using the EAGER pipeline v2.5.1^90^. They were then mapped against the mitochondrial genome (GenBank acc. no. MT663266) and the NCBI RefSeq nuclear genome (GenBank acc. no. GCA_947568905.1) using the Circular mapper and BWA in the EAGER pipeline with the default setting for aDNA reads^90^. All duplicate reads were removed using DeDup version 0.12.2, part of the EAGER pipeline, with the default options. To verify aDNA data sets, we evaluated the presence of postmortem DNA damage signatures from read alignments using mapDamage version 2.0.833.

For most analyses, we used ANGSD v0.929^47^. Unless noted otherwise, we included only genotype likelihoods with p-values < 1e- 6, mapping quality > 30, minQ > 25, and a minor allele frequency > 0.05 that were present in 20 or more individuals (option “minInd 20”). Results were visualized in the R environment v4.3.2^40^ using base functions and ggplot2 v3.5.1^91^.

### Genetic structure

We estimated individual genetic distance based on genotype likelihoods with the program PCAngsd v1.21^92^ and conducted a principal component analysis (PCA) with the ‘eigen’ function in R based on the resulting distance matrix. We also estimated individual admixture proportions for 2 to 5 ancestral populations (K) using PCAngsd. Using all sites with sufficient coverage, not only variable sites, we tested each bi-allelic site for Hardy-Weinberg-Equilibrium (HWE) and calculated the inbreeding coefficient F in each population separately with ANGSD v0.929^47^.We calculated sliding window values for Watterson’s theta, nucleotide diversity π and Tajima’s D for each population with ANGSD^47^. These values are based on the folded site frequency spectrum (SFS), which is estimated based on the complete spectrum of allele frequencies. For these analyses, we did not restrict our analyses to SNPs and did not filter out low frequency alleles (we omitted options ‘-SNP_pval 2e- 6’ and ‘-minMaf 0.05’). Windows were 50,000 bp long for all calculations, shifting in steps of 10,000 bp. These estimates should be considered exploratory as sample size and genome coverage are relatively low for these kinds of analyses^48^. We tested for significant differences in the grand mean between populations with an analysis of variance (ANOVA).

For the mitochondrial genomes, we generated consensus sequences for all sites that were covered by more than four reads in Geneious Prime v2024.0^93^. We excluded samples with more than 10% missing data across their mitochondrial genome. We aligned the consensus sequences to the reference genome using the “Map to reference” option, removed regions that were poorly covered by the majority of sequences, and exported the final alignment as a fasta file. In the R environment v4.3.2^40^, we calculated nucleotide diversity with the function ‘nuc.div’ of the package ‘PEGAS’ v1.3^94^, and reconstructed a neighbor joining tree based on genetic distances using the function ‘dist.dna’ of the package ‘ape’ v5.8^95^. We calculated confidence intervals around population-wise nucleotide diversity as 1.96 times the standard deviation divided by the square-root of the population sample size in custom R scripts. Non-overlapping confidence intervals indicate significantly different values at p = 0.05.

### Genomic signatures of adaptation

We calculated the sliding window Fst between all three population-wide comparisons with ANGSD v0.929^47^. Windows were 50,000 bp long for all calculations, shifting in steps of 10,000 bp. In such genome scans, high Fst values indicate potential regions under local selection. Significant outliers were identified with Rosner’s generalized extreme Studentized deviate test (R function ‘rosnerTest’, package ‘EnvStats’)^96,97^, where the p-value was Bonferroni corrected for the total number of Fst values tested. Using custom R scripts, we searched the annotation of the *O. edulis* genome for genes that were located on the genomic regions under potential selection. We extracted the gene names from the.gff file, and summarized the known gene functions manually.

In addition to FST outlier detection, PCAngsd was used to detect genetic markers under local adaptation while accounting for neutral population structure based on Principal Component Analysis (PCA)^49^. Using the same settings as for detecting genetic structure, we estimated the selection coefficients and p-values for each SNP for the first two principal component axes. The p-values were Bonferroni corrected for the total number of SNPs.

## Electronic supplementary material

Below is the link to the electronic supplementary material.

Supplementary Material 1

## Data Availability

The datasets used and/or analysed during the current study are available from the corresponding author on reasonable request. The assembled, annotated draft reference genome is deposited in ENA and is available under the assembly accession number GCA_963966695. The genomic data from the historical oyster shells are being analysed for other metagenomic components and will be publicly available upon completion of those analyses.
