# Supplementary material for: Museomics of an extinct European flat oyster population
Source: Sci Rep. 2025 Apr 22;15:13906. doi: 10.1038/s41598-025-96743-8 (PMC12015263; doi:10.1038/s41598-025-96743-8)
Supplement: Supplementary file 1 — Supplementary Material 1 [file 41598_2025_96743_MOESM1_ESM.pdf]

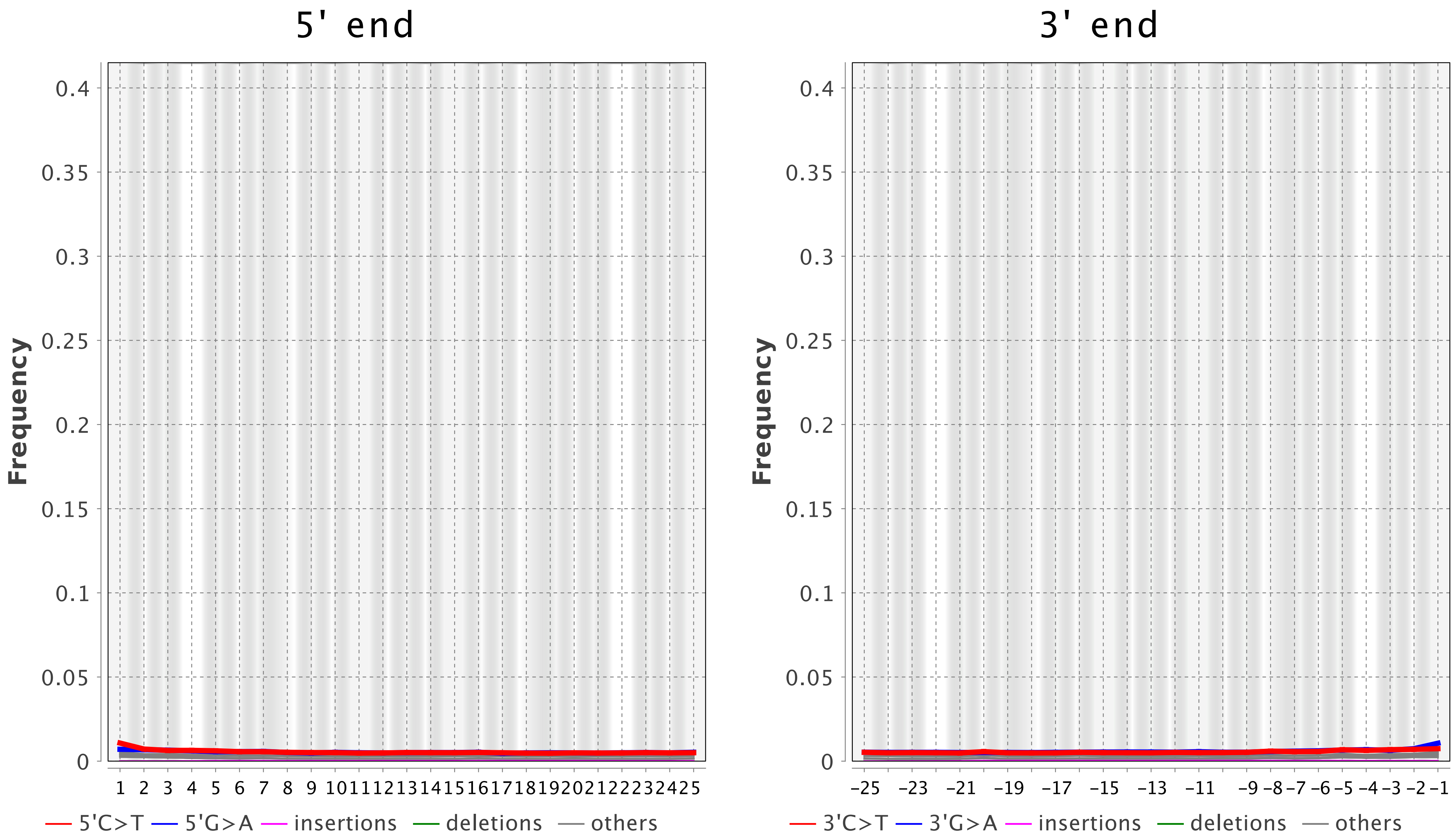

Supplementary Figure S1. DNA damage profiles for sample KAu181769 (Wadden Sea, Germany). Number of used reads: 2,758,863 (57.28% of all input reads)

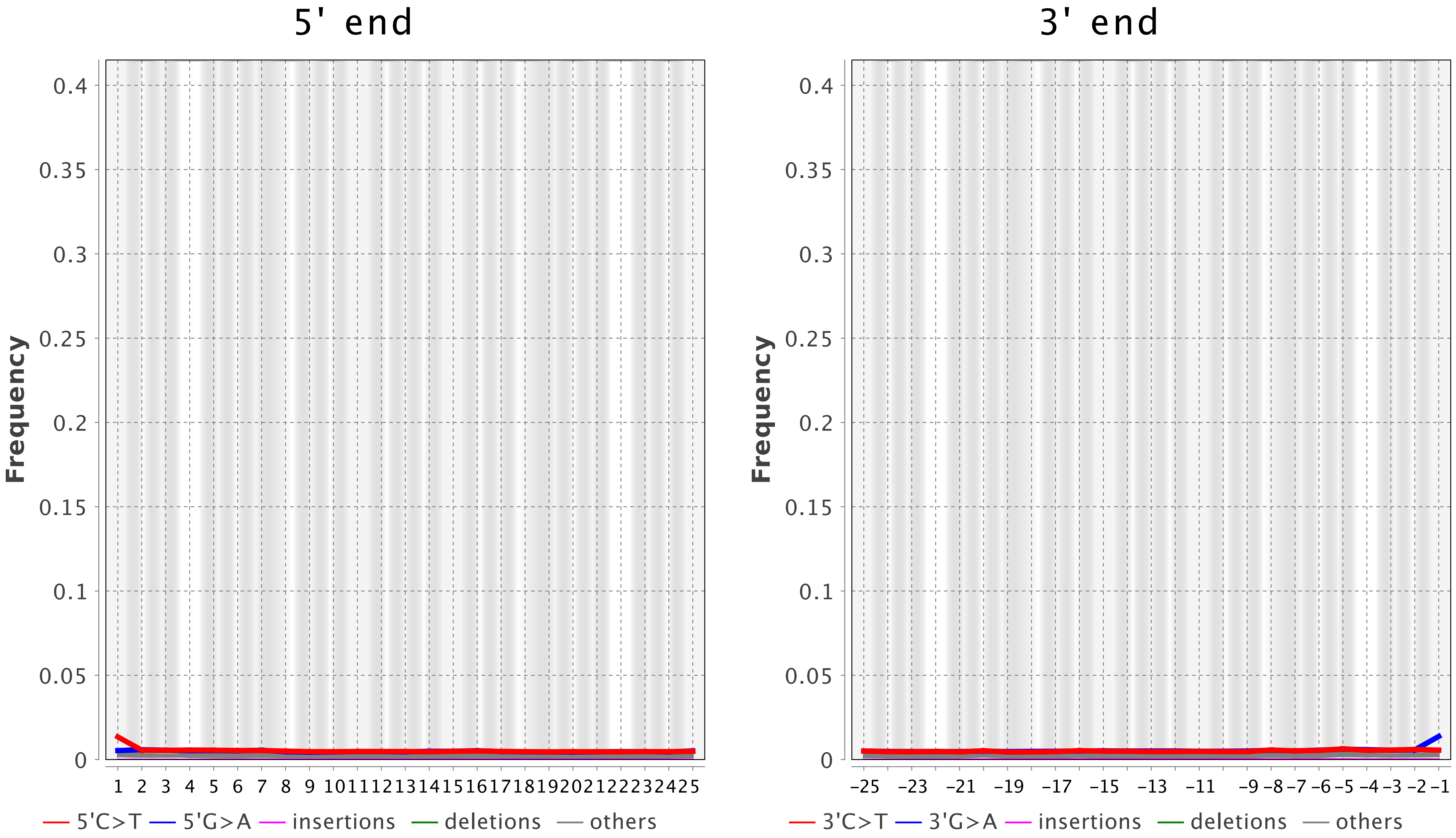

Supplementary Figure S2. DNA damage profiles for sample KAu181779 (Oosterschelde, Netherlands). Number of used reads: 3,699,622 (54.39% of all input reads)

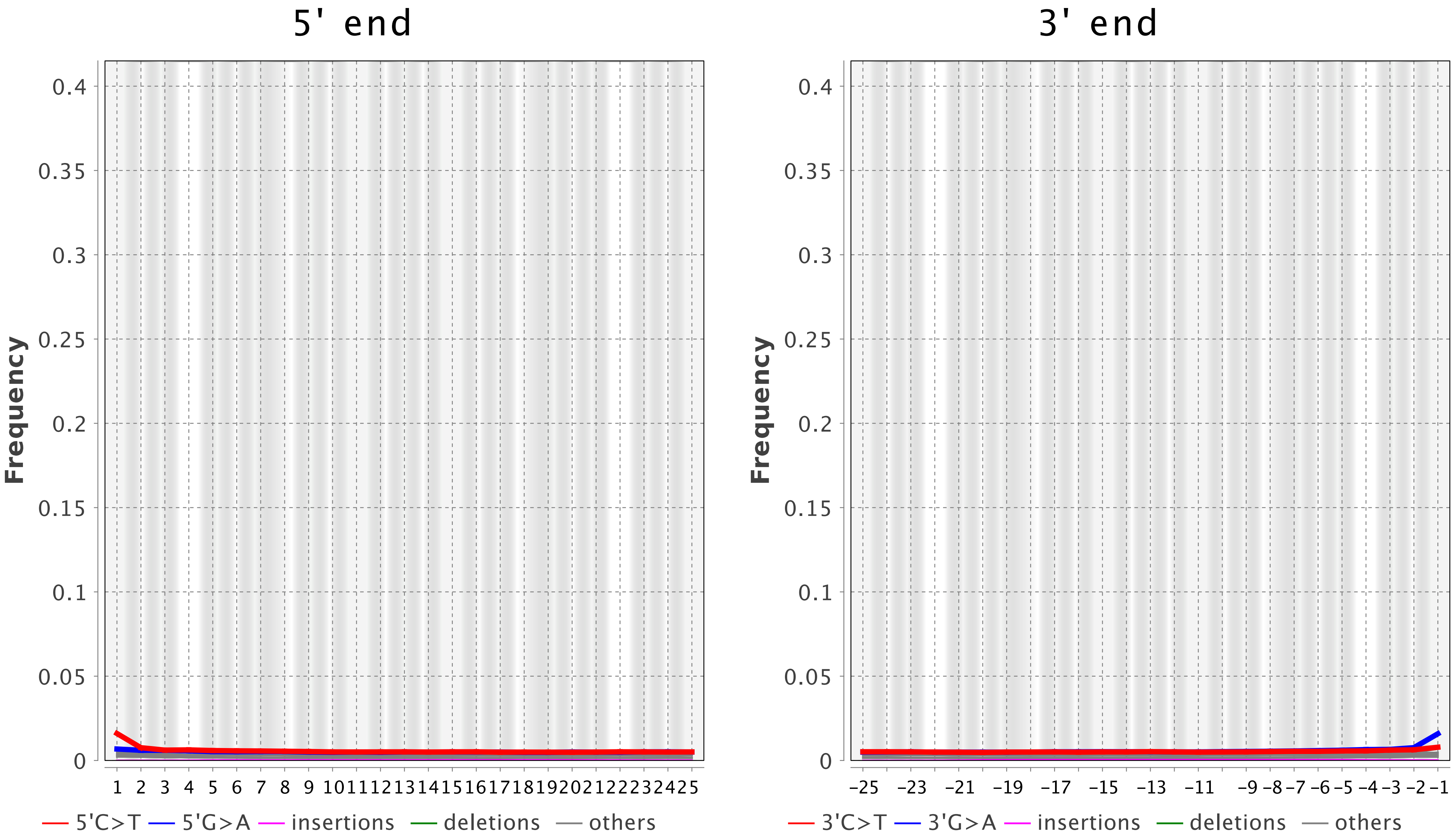

Supplementary Figure S3. DNA damage profiles for sample KAu181784 (La Tremblade, France). Number of used reads: 4,484,961(84.66% of all input reads)

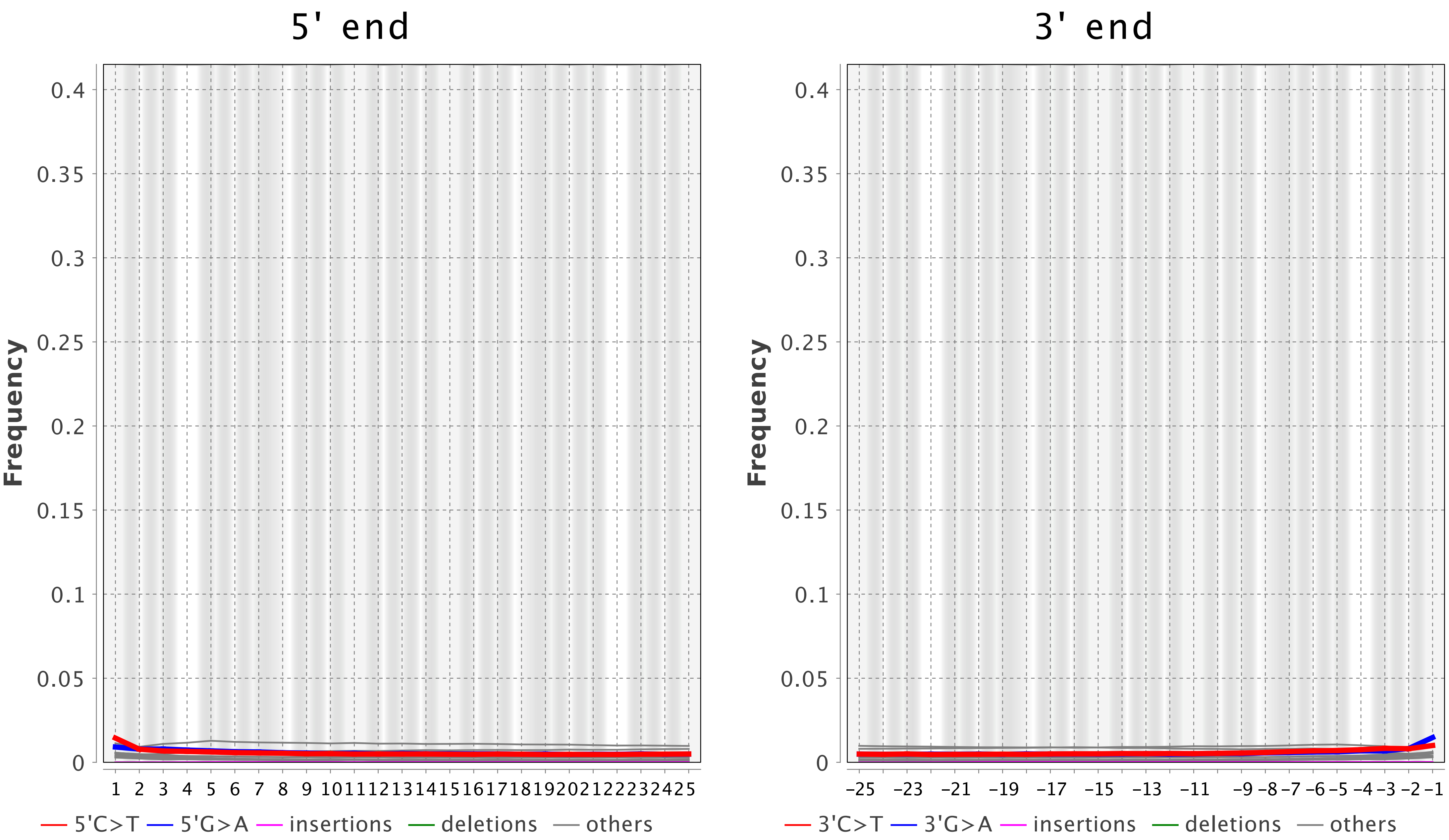

Supplementary Figure S4. DNA damage profiles for sample KAu181825 (Herne, England). Number of used reads: 4,444,151 (95.05% of all input reads)

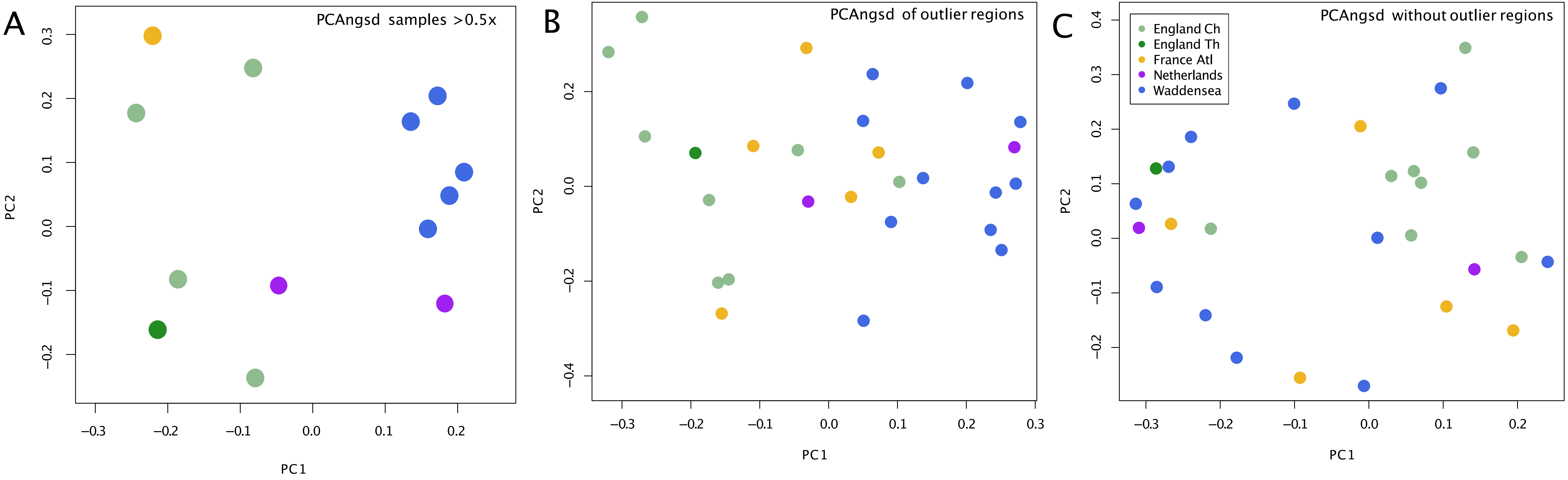

Supplementary Figure S5. PCA with A: higher coverage samples alone, B: of genomic outlier regions, C: without genomic outlier regions

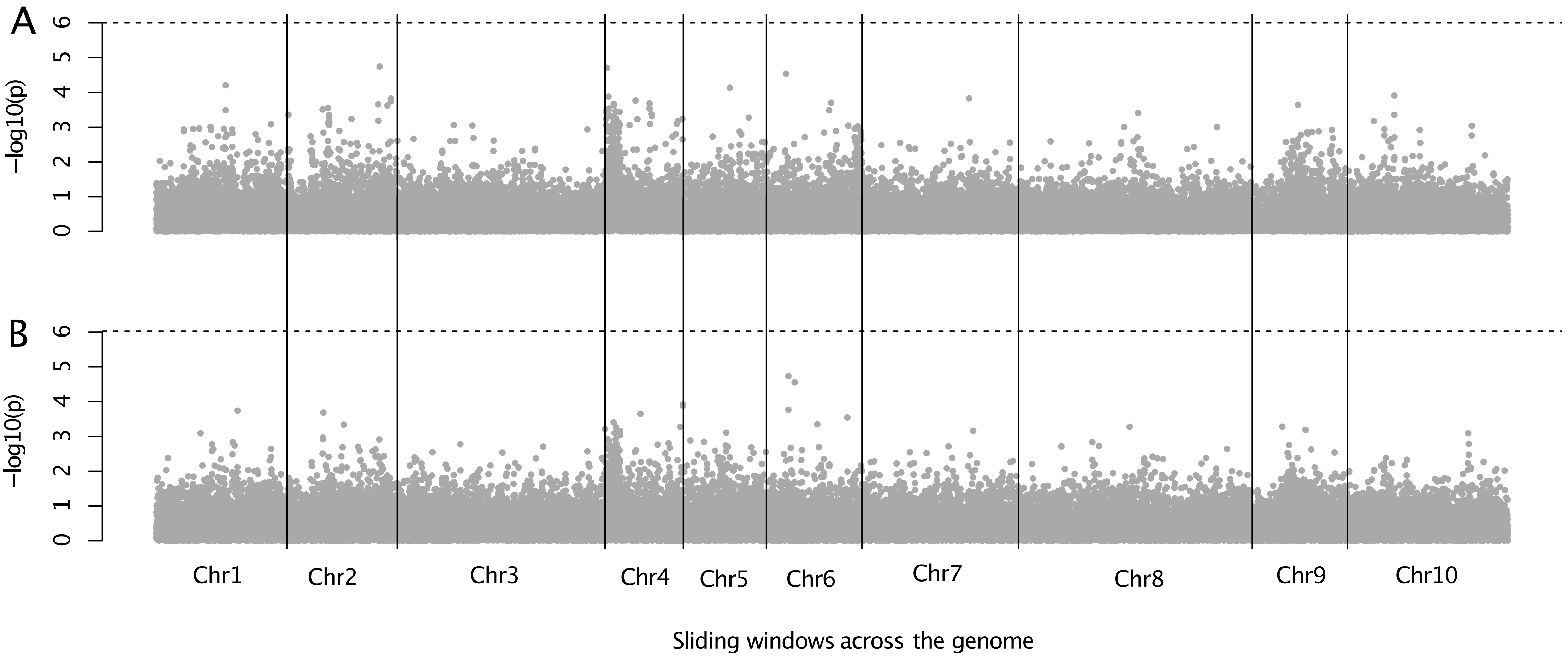

Supplementary Figure S6. PCAdapt selection scan of individual SNPs. A: principal component axis 1. B: principal component axis 2

Supplementary Table S1. Collecting information for each oyster.

ID	Collection Date	Region	Site abb.	Site	Latitude	Longitude	Description	Museum collection ID	Mitochondrial haplogroup (Hayer et al. 2021)	Included in mt analysis in this study
KAu181776	1869	GB	England Ch	Hayling	50.7855	-1.0299	Engl. Kanalkueste, Hayling Island, bei Portsmouth, Hampshire	Mo 90/1 rechts	NEA	Yes
KAu181780	1869	GB	England Ch	Hayling	50.7855	-1.0299	Engl. Kanalkueste, Hayling Island (b. Portsmouth, Hampshire), 2-	Mo 92/1 links	NEA	Yes
KAu181783	1869	GB	England Ch	Hayling	50.7855	-1.0299	Engl. Kanalkueste, Hayling Island (b. Portsmouth, Hampshire), 2-	Mo 92/4 links	-	No
KAu181827	1869	GB	England Ch	Hayling	50.7855	-1.0299	Engl. Kanalkueste, Hayling Island (b. Portsmouth, Hampshire)	Mo 91/2 rechts	-	No
KAu181828	1869	GB	England Ch	Hayling	50.7855	-1.0299	Engl. Kanalkueste, Hayling Island (b. Portsmouth, Hampshire)	Mo 91/3 rechts	SEA	Yes
KAu181829	1869	GB	England Ch	Hayling	50.7855	-1.0299	Engl. Kanalkueste, Hayling Island (b. Portsmouth, Hampshire)	Mo 91/5 links	ownHT	No
KAu181830	1869	GB	England Ch	Hayling	50.7855	-1.0299	Engl. Kanalkueste, Hayling Island (b. Portsmouth, Hampshire)	Mo 91/6 links	SEA	Yes
KAu181832	1869	GB	England Ch	Hayling	50.7855	-1.0299	Engl. Kanalkueste, Hayling Island (b. Portsmouth, Hampshire)	Mo 91/8 rechts	NS	Yes
KAu181825	1869	GB	England Th	Herne	51.5189	0.7868	Ostengl. Nordseekueste, Themse-Muendung, Herne Bay	Mo 88/1 rechts	SEA	Yes
KAu181764	1869	FR	France Atl	La Trinité sur mer	47.5777	-3.0523	Franzoesische Atlantikkueste, la Trinite	Mo 99/3 rechts	NEA	Yes
KAu181784	1869	FR	France Atl	La Tremblade	45.8449	-1.1677	Franzoesische Atlantikkueste, La Tremblade (Seudre-Muendung)	Mo 98/1 rechts	SEA	No
KAu181844	1869	FR	France Atl	Isle de Re	46.1906	-1.3073	Französische Atlantikküste, Isle du Ré (gegenueber la Rochelle)	Mo 97/4 rechts	SEA	No
KAu181845	1869	FR	France Atl	Isle de Re	46.1906	-1.3073	Franzoesische Atlantikkueste, Isle du Ré (gegenueber la Rochelle)	Mo 97/5 rechts	SEA	No
KAu181848	1869	FR	France Atl	Isle de Re	46.1906	-1.3073	Franzoesische Atlantikkueste, Isle du Re (gegenueber la Rochelle)	Mo 97/8 rechts	NEA	Yes
KAu181778	1878	NL	Netherlands	Oosterschelde	51.8848	04.0278	Oosterschelde (Holland), Kulturaustern	Mo 80/2 rechts	NEA	Yes
KAu181779	1878	NL	Netherlands	Oosterschelde	51.8848	04.0278	Oosterschelde (Holland), Kulturaustern	Mo 80/3 links	NS	Yes
KAu180235	24.03.1871	WS	Waddensea	Schleswig-Holstein	55.0560	08.4131	Schleswig-Holsteinische Westkueste	Mo 25/1	WS	Yes
KAu180257	23.05.1877	WS	Waddensea	Sylt	54.9720	08.4670	Sylt, Austernbank Huntje	Mo 58/2	WS	Yes
KAu180259	23.05.1877	WS	Waddensea	Sylt	54.9720	08.4670	Sylt, Austernbank Huntje	Mo 58/4	WS	No
KAu180268	22.05.1877	WS	Waddensea	Amrum	54.6818	8.3039	Schleswig-Holsteinische Westkueste, Austernbank "Westen	Mo 73/2	-	No
KAu180273	22.05.1877	WS	Waddensea	Amrum	54.6818	8.3039	Schleswig-Holsteinische Westkueste, Austernbank "Westen	Mo 73/7	NS	No
KAu180345	25.05.1877	WS	Waddensea	Reisby Steert	-	-	Reisby-Steert, Nordschleswigsche Westkueste (Daenemark)	Mo 47/1 rechts	WS	Yes
KAu181766	1870	WS	Waddensea	Amrum	54.6818	8.3039	Schleswig-Holsteinische Westkueste, Amrum, 6-jaehrig	Mo 72/1 rechts	WS	Yes
KAu181769	1870	WS	Waddensea	Schleswig-Holstein	55.0560	8.4131	Schleswig-Holsteinische Westkueste	Mo 39/1 rechts	NS	Yes
KAu181772	1879	WS	Waddensea		55.0560	8.4131	Schleswig-Holsteinische Austernbaenke, vom Cirrenwurm	Mo 7/3 rechts	WS	Yes
KAu181773	1879	WS	Waddensea	Schleswig-Holstein	55.0560	8.4131	Schleswig-Holsteinische Austernbaenke, vom Cirrenwurm	Mo 7/4 rechts	NS	Yes

Supplementary Table S2. Description of collection sites.

Locality	Marine unit	Exact locality	Country	Approx. geographic coordinates	Sample size	Oyster collection site description from Möbius (1870)	Notes
Wadden Sea	NorthSea	Reisby–Steert	Denmark	55.224002, 8.570119	1	N/A	
Wadden Sea	NorthSea	Schleswig–Holstein	Germany	N/A	4	N/A	
Wadden Sea	NorthSea	Amrum	Germany	N/A	1	N/A	
Wadden Sea	NorthSea	Oyster bed "Westen Amrum"	Germany	54.665706, 8.284123	2	N/A	
Wadden Sea	NorthSea	Sylt	Germany	N/A	1	N/A	
Wadden Sea	NorthSea	Oyster bed "Huntje" near Sylt	Germany	54.995381, 8.46765777	2	N/A	
Netherlands	NorthSea	Oosterschelde	Holland	51.493309, 4.116461	2	"Kulturaustern"	Collected oysters were possibly not native to the collecting site
Engl. Thames	NorthSea	Herne Bay, mouth of the Thames	England	51.384079, 1.128908	1	Seed oysters from Wales, Ireland and Ostend (EngliSchleswig–Holstein Channel), as well as local oysters ("Natives")	Collected oysters were possibly not native to the collecting site
Engl. Channel	EngliSchleswig–Holstein Channel	Hayling Island near Portsmouth, HampSchleswig–Holsteinire	England	50.828068, –0.986566	7	Spat from surrounding natural oyster beds is used for seed oysters	
France Atl.	Bay of Biscay	La Trinity	France	47.578325, –3.019161	1	Spat from surrounding natural oyster beds is used for seed oysters	
France Atl.	Bay of Biscay	Isle de Ré	France	46.227113, –1.475321	3	Spat from surrounding natural oyster beds is used for seed oysters	
France Atl.	Bay of Biscay	La Tremblade (mouth of the Seudre)	Fance	45.798205, –1.187801	1	Fattening of young oysters from Cancale (EngliSchleswig–Holstein Channel) and Brittany	Collected oysters were possibly not native to the collecting site
France Med.	Mediterranean	Toulon	France	43.107761, 5.910555	3	Schleswig–Holsteinells of seed oysters from the Mediterranean and Atlantic Ocean	Collected oysters were possibly not native to the collecting site

Supplementary Table S3. Nuclear genome assembly summary

Sample	Region	#_duplicates	duplicate_proportion_(%)	bp_ref	bp_covered	#_reads_rmdup	read_length_rmdup_(bp)	rmdup_cov>=1x_(%)	rmdup_cov>=2x_(%)	rmdup_cov>=3x_(%)	rmdup_cov>=4x_(%)	mean(DP)_cov	mean(DP)_all
KAu181764	FR	13747	0	967083865	309061765	4958566	86.0669 (25–192)	31.9581	2.0314	0.573002	0.222285	1.38039	0.441147
KAu181784	FR	93873	1	967083865	349067509	6439881	78.3719 (25–192)	36.0949	2.91171	0.882204	0.342996	1.44539	0.521711
KAu181844	FR	136967	4	967083865	215189447	3735183	74.0087 (25–192)	22.2514	0.899901	0.261122	0.115254	1.2842	0.285753
KAu181845	FR	3687	0	967083865	136938985	2198875	72.1682 (25–192)	14.16	0.23468	0.0648548	0.0333458	1.1583	0.164015
KAu181848	FR	31062	1	967083865	272013879	5057013	70.6937 (25–192)	28.1272	1.33036	0.353742	0.141828	1.31378	0.36953
KAu181776	GB	998390	4	967083865	663283558	24912352	65.0731 (25–192)	68.5859	23.7662	12.4534	6.44158	2.44342	1.67584
KAu181780	GB	426275	4	967083865	464489863	10929500	76.5552 (25–192)	48.0299	7.87668	3.15585	1.39356	1.80078	0.864915
KAu181783	GB	67178	1	967083865	256235315	4785545	70.8234 (25–192)	26.4957	1.30844	0.368973	0.151353	1.32226	0.350341
KAu181825	GB	489687	3	967083865	522611430	18320136	58.0078 (25–192)	54.0399	12.0306	5.80434	3.02484	2.0329	1.09858
KAu181827	GB	48818	4	967083865	80672599	1198160	76.1385 (25–192)	8.34184	0.104785	0.0360904	0.0220841	1.13041	0.0942968
KAu181828	GB	129797	1	967083865	534904196	13636516	76.7111 (25–192)	55.311	12.1599	5.40015	2.51709	1.9551	1.08138
KAu181829	GB	61020	2	967083865	173942542	2876406	73.675 (25–192)	17.9863	0.475796	0.131764	0.0620287	1.21784	0.219045
KAu181830	GB	28626	1	967083865	149828867	2620005	67.2757 (25–192)	15.4929	0.298036	0.0800169	0.0385181	1.17599	0.182194
KAu181832	GB	52651	3	967083865	105031060	1661594	72.9111 (25–192)	10.8606	0.161171	0.0509581	0.0284519	1.15294	0.125216
KAu181778	NL	453536	3	967083865	533769933	14347318	72.0879 (25–192)	55.1938	11.7531	5.10073	2.33907	1.93712	1.06917
KAu181779	NL	674778	2	967083865	719026119	27331785	89.6729 (25–192)	74.3499	37.4507	25.1454	16.8137	3.40787	2.53375
KAu180235	WS	115294	1	967083865	376317955	7583057	77.5098 (25–192)	38.9126	4.28085	1.50951	0.631003	1.56145	0.607601
KAu180257	WS	121158	2	967083865	302064290	5959204	72.6835 (25–192)	31.2345	2.27959	0.762145	0.338932	1.43355	0.447764
KAu180259	WS	29165	2	967083865	124267581	1661573	87.0494 (25–192)	12.8497	0.206051	0.0560193	0.0289529	1.16359	0.149518
KAu180268	WS	24761	2	967083865	86415665	1219449	81.123 (25–192)	8.9357	0.115242	0.0368458	0.0209849	1.14441	0.102261
KAu180273	WS	107624	7	967083865	69462066	1380559	70.6308 (25–192)	7.18263	0.0926613	0.0517298	0.042736	1.40342	0.100802
KAu180345	WS	211549	2	967083865	426791680	10290916	83.5483 (25–192)	44.1318	8.33506	4.42805	2.68376	2.01407	0.888846
KAu181766	WS	304616	9	967083865	171355055	3163061	68.1491 (25–192)	17.7187	0.607382	0.188448	0.0909452	1.2575	0.222813
KAu181769	WS	406106	2	967083865	647577716	17529652	84.9482 (25–192)	66.9619	21.4449	10.4833	5.0391	2.29888	1.53937
KAu181772	WS	48270	1	967083865	348364748	6176550	80.0183 (25–192)	36.0222	2.67415	0.757171	0.283663	1.41829	0.5109
KAu181773	WS	407128	5	967083865	380571567	7786814	74.7528 (25–192)	39.3525	3.99069	1.34136	0.545312	1.52903	0.601713

Supplementary Table S4. Functions of outlier genes detected in genomic regions of high Fst between the Wadden Sea population and the English and French population.

Dbxref	description	gbkey	gene	gene_biotype	chromosome_r	chromosome_r	Function	Source	Potential function in oysters
GeneID:125663622	opsin-1-like	Gene	LOC125663622	protein_coding	1	NC_079164.1	green cone photopigment or medium-wavelength sensitive opsin	https://www.ncbi.nlm.nih.gov/gene/2652	
GeneID:125662480	uncharacterized LOC125662480	Gene	LOC125662480	protein_coding	1	NC_079164.1			
GeneID:130047952	uncharacterized LOC130047952	Gene	LOC130047952	lncRNA	1	NC_079164.1			
GeneID:125663978	paraplegin-like	Gene	LOC125663978	protein_coding	1	NC_079164.1	diverse cellular processes including membrane trafficking, intracellular motility, organelle biogenesis, protein folding, and proteolysis. The SPG7 protein is a transmembrane protein	https://en.wikipedia.org/wiki/Paraplegin	Hypoxia stress in animals - but apparently not oysters (Steffen et al. 2020)
GeneID:125652215	trafficking protein particle complex subunit 6b-like	Gene	LOC125652215	protein_coding	5	NC_079168.1	Inter-organelle traffic, involved in the early development of neural circuitry	https://www.uniprot.org/uniprotkb/Q86SZ2/entry	
GeneID:125648923	sodium-dependent phosphate transport protein 2B-like	Gene	LOC125648923	protein_coding	5	NC_079168.1	Involved in actively transporting phosphate into cells via Na+ cotransport	https://www.uniprot.org/uniprotkb/O95436/entry	Osmoregulation (Zhao et al. 2016)
GeneID:125648921	sodium-dependent phosphate transport protein 2B-like	Gene	LOC125648921	protein_coding	5	NC_079168.1	Involved in actively transporting phosphate into cells via Na+ cotransport	https://www.uniprot.org/uniprotkb/O95436/entry	Osmoregulation (Zhao et al. 2016)
GeneID:125648922	sodium-dependent phosphate transport protein 2B-like	Gene	LOC125648922	protein_coding	5	NC_079168.1	Involved in actively transporting phosphate into cells via Na+ cotransport	https://www.uniprot.org/uniprotkb/O95436/entry	Osmoregulation (Zhao et al. 2016)
GeneID:125652196	proteasome subunit alpha type-3-like	Gene	LOC125652196	protein_coding	5	NC_079168.1	Involved in the proteolytic degradation of most intracellular proteins	https://www.uniprot.org/uniprotkb/P25788/entry	Cell apoptosis (Zhang et al. 2016)
GeneID:125651075	calcium permeable stress-gated cation channel 1-like	Gene	LOC125651075	protein_coding	5	NC_079168.1	Acts as an osmosensitive calcium-permeable cation channel.	https://www.uniprot.org/uniprotkb/Q06538/entry	Osmoregulation (Zhang et al. 2016)
GeneID:125651496	serum response factor-like	Gene	LOC125651496	protein_coding	5	NC_079168.1	SRF is important during the development of the embryo, as it has been linked to the formation of mesoderm.	https://en.wikipedia.org/wiki/Serum_response_factor	
GeneID:125650347	glutaminyl-peptide cyclotransferase-like	Gene	LOC125650347	protein_coding	5	NC_079168.1	Biosynthesis of pyroglutamyl peptides	https://www.uniprot.org/uniprotkb/Q16769/entry	
GeneID:125651749	poly(A) polymerase type 3-like	Gene	LOC125651749	protein_coding	5	NC_079168.1	Polyadenylation of transcripts	https://www.ncbi.nlm.nih.gov/pmc/articles/PMC2525947/	
GeneID:125651753	seipin-like	Gene	LOC125651753	protein_coding	5	NC_079168.1	Membrane protein in the ER, central regulation of energy homeostasis, lipid catabolism (essential for adipocyte differentiation), lipid storage and lipid droplet maintenance	https://en.wikipedia.org/wiki/Seipin	Stress response (Zhang et al. 2016)
GeneID:130054660	uncharacterized LOC130054660	Gene	LOC130054660	lncRNA	5	NC_079168.1			
GeneID:125651755	menin-like	Gene	LOC125651755	protein_coding	5	NC_079168.1	Function unknown, putative tumor suppressor	https://en.wikipedia.org/wiki/MEN1	Immune response (Li et al. 2016)
GeneID:125651750	putative lipid scramblase CLPTM1	Gene	LOC125651750	protein_coding	5	NC_079168.1	Translocating lipids in membranes from one leaflet to the other one. Required for efficient glycosylphosphatidylinositol (GPI) inositol deacylation in the ER	https://www.uniprot.org/uniprotkb/O96005/entry	Possible role in general stress response, e.g. to hypoxia, heat (Zhang et al. 2016)
GeneID:130050512	uncharacterized LOC130050512	Gene	LOC130050512	protein_coding	5	NC_079168.1			
GeneID:125662581	uncharacterized LOC125662581	Gene	LOC125662581	protein_coding	5	NC_079168.1			
GeneID:125663628	uncharacterized LOC125663628	Gene	LOC125663628	protein_coding	5	NC_079168.1			
GeneID:125652017	probable G-protein coupled receptor 139	Gene	LOC125652017	protein_coding	5	NC_079168.1	Research has shown that mice with loss of GCP139 experience schizophrenia-like symptomatology	https://en.wikipedia.org/wiki/GPR139	Possible role in immune response, deduced from functions of similar genes (Jia et al. 2018)
GeneID:125652213	pinin-like	Gene	LOC125652213	protein_coding	5	NC_079168.1	Enables RNA binding activity. Predicted to be involved in cell adhesion and mRNA splicing, via spliceosome. Predicted to act upstream of or within cell-cell adhesion.	https://www.genecards.org/cgi-bin/carddisp.pl?gene=PNN	
GeneID:125651440	2-aminoethylphosphonate--pyruvatetransaminase-like	Gene	LOC125651440	protein_coding	5	NC_079168.1	Transfer of nitrogenous groups, participates in aminophosphonate metabolism	https://www.ebi.ac.uk/interpro/entry/InterPro/IPR012703/	Stress response (Zhang et al. 2016)
GeneID:130054664	neuronal acetylcholine receptor subunit alpha-9-like	Gene	LOC130054664	protein_coding	5	NC_079168.1	Probable role in the modulation of auditory stimuli. Agonist binding induces a conformation change that leads to the opening of an ion-conducting channel across the plasma membrane	https://www.uniprot.org/uniprotkb/Q9UGM1/entry	Osmoregulation (Xiao et al. 2018)
GeneID:125660324	uncharacterized LOC125660324	Gene	LOC125660324	protein_coding	9	NC_079172.1			

Supplementary Information S1. Preliminary analysis based on mappings against the draft genome

Rationale: As mentioned in the main manuscript, we first conducted our historical population genomic analyses based on mappings against a fragmented draft genome of Ostrea edulis (described in teh manuscript). Here we present these initial findings. They show a large number of outlier regions that we interpreted as a sign of local adaptation. However, these outlier regions were no longer as pronounced when re-analysing the data mapped against a chromosome-level genome assembly (Gundappa et al. 2022). The overall population genetic structure, on the other hand, was less impacted by the choice of reference genome.

Material and methods

Whole genome shotgun sequencing, quality control and mapping

Reads were mapped against the draft genome we generated for Ostrea edulis as described in the main manuscript. Genotype likelihoods were estimated with the same filters as in the main manuscript.

Genetic structure

We estimated individual genetic distance using the single read sampling approach and conducted a principal component analysis (PCA) with the 'eigen' function in R based on the resulting distance matrix. We also estimated individual admixture proportions for 2 to 5 ancestral populations (K) using NGSAdmix (Skotte et al., 2013). To infer the maximal value of K, we replicated the analysis 10 times and estimated delta K (Evanno et al., 2005) using R scripts available in the online version of the Marine Genomics course of the University of California Davis (https://baylab.github.io/MarineGenomics/week-9--population-structure-using-ngsadmix.html#week-9--population-structure-using-ngsadmix).We plotted the results of the replicate with the highest log likelihood. We tested each bi-allelic site for Hardy-Weinberg-Equilibrium(HWE) and calculated the inbreeding coefficient F in each population separately.

Genomic signatures of local adaptation

To detect regions under potential local adaptation, we compared the Wadden Sea population with a combined French-British population. We combined French and British samples based on the results of the PCA. First, we calculated sliding window values for theta and Tajima's D for each population. Genomic regions of particularly low theta and negative Tajima's D values in the Wadden Sea population that do not show the same pattern in the French-British population may have undergone recent selective sweeps (Tajima, 1989). Windows were 50,000 bp long for all calculations, shifting in steps of 10,000 bp. However, as the genome coverage was low and not even throughout the alignment, the number of sites that were available for the calculation of the different statistics varied between sliding windows. We omitted windows with fewer than 10,000 usable sites. Outlier values were determined by linear regression between Tajima's D values from each population and calculation of Cook's distance in R (functions 'lm' and 'cooksd'). The same approach was used for theta. Furthermore, we calculated sliding window Fst between the two populations. In such genome scans, high Fst values indicate potential regions that are under local selection. Significant outliers were identified with Rosner's generalized extreme Studentized deviate test (Rosner, 1975), where the p-value was Bonferroni corrected for the total number of Fst values tested. We then identified the genomic scaffolds that contained high Fst, low theta as well as negative Tajima's D values as the best candidate targets of local selection.

Results

Whole genome shotgun sequencing, quality control and mapping

Between 9.357% and 83.352% (median = 36.488%) of the reads mapped to the draft nuclear genome of O. edulis. After combining the runs for each sample, median genome coverage was 0.4478x, and ranged from 0.1008 to 2.5337x. A total of 243,005 sites passed our genotype filters and were used in the subsequent analyses.

Genetic structure

In the PCA, genomes of the now-extinct Wadden Sea population clustered tightly with each other, while the British and French oysters were less differentiated (Fig. 1A). The two Dutch oysters clustered with the Wadden Sea and French-British populations, respectively (Fig. 2A). For the admixture analysis, K=3 had the highest delta K value (1135.6372), followed by K=5 (927.8341), K=2 (912.6502) and K=4 (895.1018). The admixture analysis were concordant with the PCA results: the Wadden Sea oysters belonged predominantly to the first ancestral population, the British oysters to the second and third ancestral population, and the French oysters to the third ancestral population (Fig. 1B,C). We tested for HWE and inbreeding in the Wadden Sea and French-British population separately. Of 634,136 SNPs, 29,481 (4.65%) had a significant inbreeding coefficient F and departure from HWE in the French-British population, and 18,875 (2.97%) in the Wadden Sea population. Only four of these SNPs deviated from HWE in both populations.

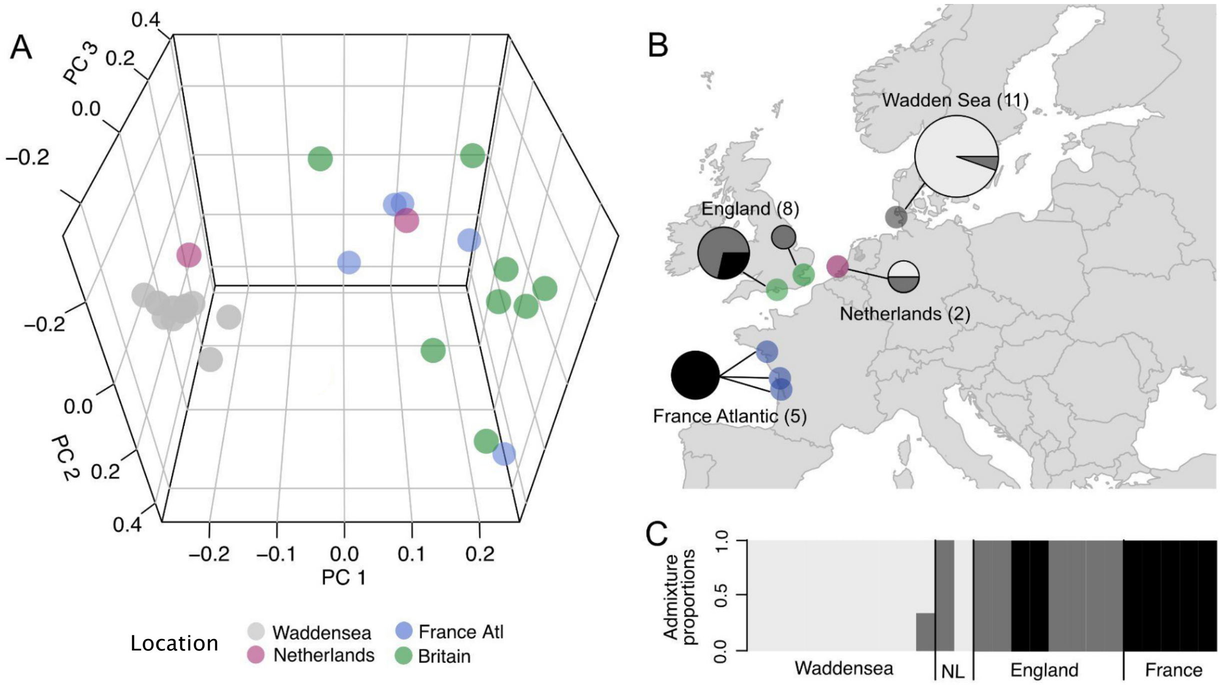

Figure 1. Genomic structure of European oysters collected at the end of the 19th century. A: Sampling localities with sampling sizes (n) and results of admixture analysis summarized per sampling locality. B: Admixture plot. Each bar is one individual, and the colors denote the ancestral populations with

K=3. C: PCA plot. Samples are colored based on their sampling locality. Note that the Limfjord oyster (the individual used for the reference genome) was contemporary. NL: Netherlands.

Genomic signatures of local adaptation

For all genome scans, we compared the Wadden Sea population to the combined French-British population based on the results of the admixture analysis. The genome-wide median of Tajima's D for the Wadden Sea population was -0.425, and -1.013 for the French-British population (Fig. 2A). Plotting Tajima's D values for each population against each other revealed a cluster of 818 values that were either relatively low in the Wadden Sea population, or relatively high in the French-British population (Fig. 2A). These outliers clustered on 46 genomic scaffolds. Genome-wide Watterson's theta was 324.74 for the French-British population, and 209.03 for the Wadden Sea population. Plotting Watterson's theta values between populations revealed a similar cluster of outliers as for Tajima's D, but with a less clear separation to the overall distribution (Fig. 2B).

The genome-wide unweighted Fst was 0.030766, and the weighted Fst was 0.052661. The distribution of sliding window Fst values was bimodal, with the majority of values centering around the genome-wide median (Fig. 2E). However, of the 47,703 sliding windows, 1,197 had Fst values that were significant outliers, centering around 0.22 (Fig. 2E). Of the 14,796 genomic fragments that make up the draft genome, the high Fst values clustered on 49 of these fragments. The outliers were exclusively found on fragments of intermediate genetic diversity in the Wadden Sea population (Fig. 2C) and relatively high genetic diversity in the French-British populations (Fig. 2D). This means that the genetic diversity surrounding these highly differentiated regions was lower in the Wadden Sea population than in the French-British population, indicative of selective sweeps in the Wadden Sea population. Outliers from Fst, Tajima's D and theta occurred concomitantly on 40 genomic scaffolds that were between 70,975 and 1,041,195bp long. These scaffolds contain the strongest candidates for local adaptation.

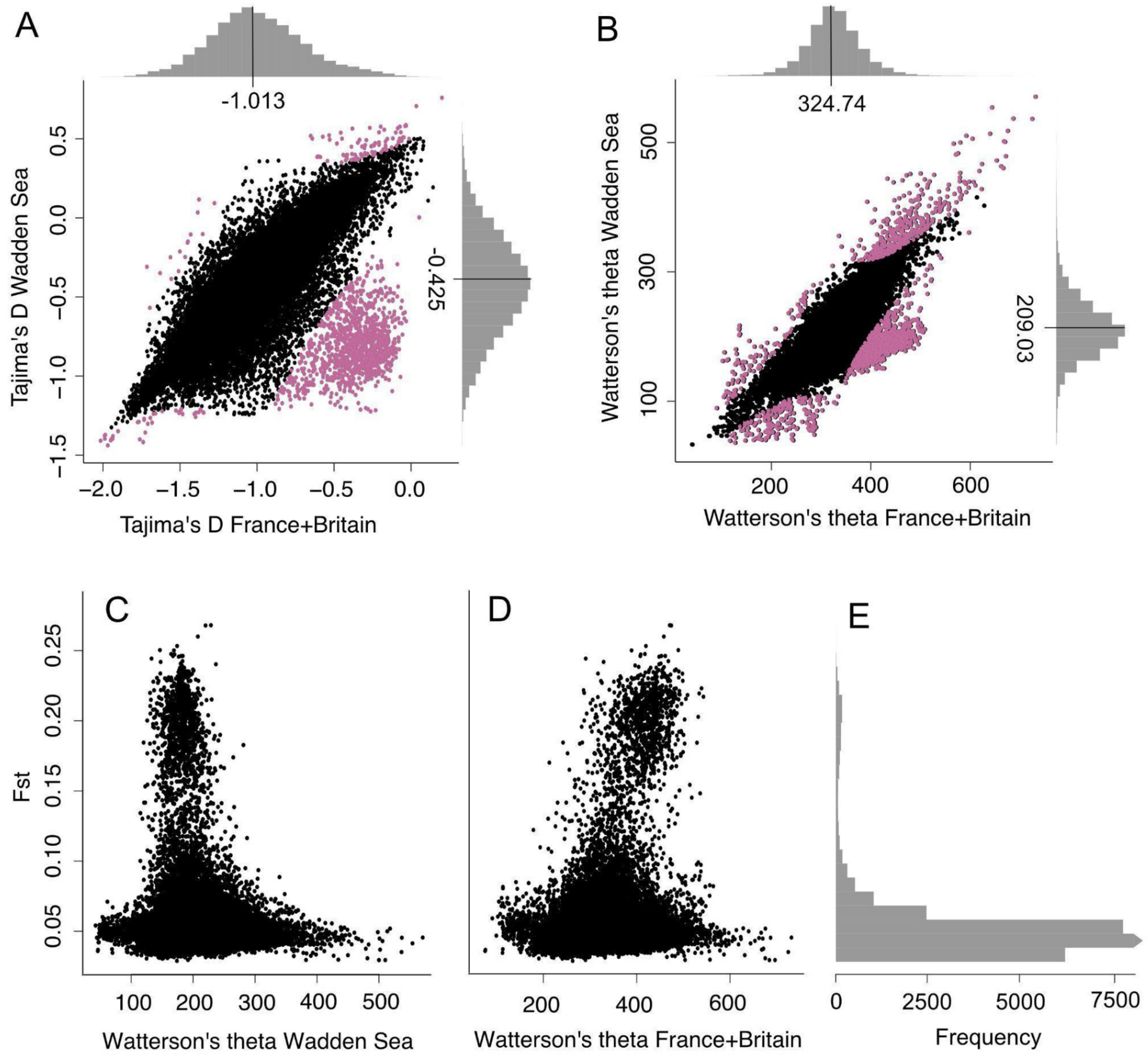

Figure 2. Genome scans for local adaptation signatures in the historical Ostrea edulis genomes. A: Sliding window Tajima's D values from the Wadden Sea population plotted against the values for the combined French and British population. B: Sliding window Wadderson's theta for the same populations. C: Genetic differentiation(Fst) of genomic windows plotted against Watterson's theta for the Wadden Sea population. D: Genetic differentiation for sliding windows plotted against Watterson's theta for the combined French-British population. E: Distribution of sliding window Fst values.

References

Adey, W. H., & Steneck, R. S. (2001). Thermogeography over time creates biogeographic regions: A temperature/space/time-integrated model and an abundance-weighted test for benthic marine algae. Journal of Phycology, 37(5), 677–698.

Beck, M. W., Brumbaugh, R. D., Airoldi, L., Carranza, A., Coen, L. D., Crawford, C., Defeo, O., Edgar, G. J., Hancock, B., & Kay, M. C. (2011). Oyster reefs at risk and

recommendations for conservation, restoration, and management. Bioscience, 61(2), 107-116.

Bi, K., Linderoth, T., Vanderpool, D., Good, J. M., Nielsen, R., & Moritz, C. (2013). Unlocking the vault: Next-generation museum population genomics. Molecular Ecology, 22(24), 6018–6032. https://doi.org/10.1111/mec.12516

Biosafety Unit. (2001). Status and Trends of Global Biodiversity. In Global Biodiversity Outlook (1st ed.). Secretariat of the Convention on Biological Diversity. https://www.cbd.int/gbo1/

Bouma, S., & Lengkeek, W. (2012). Benthic communities on hard substrates of the offshore wind farm. Rep by Bur Waardenbg Bv Noordzeewind, 84.

Bromley, C., McGonigle, C., Ashton, E. C., & Roberts, D. (2016). Bad moves: Pros and cons of moving oysters - A case study of global translocations of Ostrea edulis Linnaeus, 1758 (Mollusca: Bivalvia). Ocean & Coastal Management, 122, 103-115. https://doi.org/10.1016/j.ocecoaman.2015.12.012

Brown, J., Hill, A., Fernand, L., & Horsburgh, K. (1999). Observations of a seasonal jet-like circulation at the central North Sea cold pool margin. Estuarine, Coastal and Shelf Science, 48(3), 343-355.

Burns, P. A., Rowe, K. C., Parrott, M. L., & Roycroft, E. (2023). Population genomics of decline and local extinction in the endangered Australian Pookila. Biological Conservation, 284, 110183.

Cameron, S. A., Lozier, J. D., Strange, J. P., Koch, J. B., Cordes, N., Solter, L. F., & Griswold, T. L. (2011). Patterns of widespread decline in North American bumble bees. Proceedings of the National Academy of Sciences, 108(2), 662. https://doi.org/10.1073/pnas.1014743108

Cardinale, B. J., Duffy, J. E., Gonzalez, A., Hooper, D. U., Perrings, C., Venail, P., Narwani, A., Mace, G. M., Tilman, D., & Wardle, D. A. (2012). Biodiversity loss and its impact on humanity. Nature, 486(7401), 59–67.

Cavanaugh, K. C., Dangremond, E. M., Doughty, C. L., Williams, A. P., Parker, J. D., Hayes, M. A., Rodriguez, W., & Feller, I. C. (2019). Climate-driven regime shifts in a mangrove-salt marsh ecotone over the past 250 years. Proceedings of the National Academy of Sciences, 201902181. https://doi.org/10.1073/pnas.1902181116

Ceballos, G., & Ehrlich, P. R. (2002). Mammal population losses and the extinction crisis. Science, 296(5569), 904-907.

Chen, N. (2004). Using RepeatMasker to identify repetitive elements in genomic sequences. In Current protocols in bioinformatics.

Collen, B., Loh, J., Whitmee, S., McRAE, L., Amin, R., & Baillie, J. E. (2009). Monitoringchange in vertebrate abundance: The Living Planet Index. Conservation Biology, 23(2), 317–327.

Collen, B., McRae, L., Deinet, S., De Palma, A., Carranza, T., Cooper, N., Loh, J., & Baillie, J. E. (2011). Predicting how populations decline to extinction. Philosophical Transactions of the Royal Society B: Biological Sciences, 366(1577), 2577–2586.

Cunningham, F., Allen, J. E., Allen, J., Alvarez-Jarreta, J., Amode, M. R., Armean, I. M., Austine-Orimoloye, O., Azov, A. G., Barnes, I., & Bennett, R. (2022). Ensembl 2022. Nucleic Acids Research, 50(D1), D988–D995.

Delibes-Mateos, M., Smith, A. T., Slobodchikoff, C. N., & Swenson, J. E. (2011). The paradox of keystone species persecuted as pests: A call for the conservation of abundant small mammals in their native range. Biological Conservation, 144(5), 1335–1346.

Di Tommaso, P., Chatzou, M., Floden, E. W., Barja, P. P., Palumbo, E., & Notredame, C. (2017). Nextflow enables reproducible computational workflows. Nature Biotechnology, 35(4), 316–319.

Diaz-Almela, E., Boudry, P., Launey, S., Bonhomme, F., & Lapegue, S. (2004). Reduced female gene flow in the European flat oyster Ostrea edulis. Journal of Heredity, 95(6), 510–516.

Dirzo, R., Young, H. S., Galetti, M., Ceballos, G., Isaac, N. J., & Collen, B. (2014). Defaunation in the Anthropocene. Science, 345(6195), 401–406.

DONG Energy, A., Vattenfall, The Danish Energy Authority, & The Danish Forest and Nature Agency. (2006). Danish offshore wind-keyenvironmental issues. https://tethys.pnnl.gov/sites/default/files/publications/Danish_Offshore_Wind_Key_Environmental_Issues.pdf

Evanno, G., Regnaut, S., & Goudet, J. (2005). Detecting the number of clusters of individuals using the software structure: A simulation study. Molecular Ecology, 14, 2611–2620.

Eyton, T. C. (1858). A history of the oyster and the oyster fisheries. JohnvanVoorst, Paternoster Row. https://books.google.de/books?hl=en&lr=&id=KDoDAAAAQAAJ&oi=fnd&pg=PR1&ots=g5Mn_QpjYG&sig=yTGRBAsMW6ZwE1rll7L8FfTtzO4&redir_esc=y#v=onepage&q&f=false

Frankham, R. (2005). Genetics and extinction. Biological Conservation, 126(2), 131–140.

Garner, A., Rachlow, J. L., & Hicks, J. F. (2005). Patterns of genetic diversity and its loss in mammalian populations. Conservation Biology, 19(4), 1215–1221.

Geburzi, J. C., Heuer, N., Homberger, L., Kabus, J., Moesges, Z., Ovenbeck, K., Brandis, D., &

Ewers, C. (2022). An environmental gradient dominates ecological and genetic differentiationof marine invertebrates between the North and Baltic Sea. Ecology and Evolution, 12(5), e8868.

Gercken, J., & Schmidt, A. (2014). Current status of the European Oyster (Ostrea edulis)and possibilities for restoration in the German North Sea. Neu Broderstorf.

Gotoh, O. (2008). Direct mapping and alignment of protein sequences onto genomic sequence. Bioinformatics, 24(21), 2438–2444.

Gutierrez, A. P., Turner, F., Gharbi, K., Talbot, R., Lowe, N. R., Peñaloza, C., McCullough, M., Prodöhl, P. A., Bean, T. P., & Houston, R. D. (2017). Development of a Medium Density Combined-Species SNP Array for Pacific and European Oysters (Crassostrea gigas and Ostrea edulis). G3: Genes|Genomes|Genetics, 7(7), 2209. https://doi.org/10.1534/g3.117.041780

Haas, B. J., Salzberg, S. L., Zhu, W., Pertea, M., Allen, J. E., Orvis, J., White, O., Buell, C. R., & Wortman, J . R. (2008). Automated eukaryotic gene structure annotation using EVidenceModeler and the Program to Assemble Spliced Alignments. Genome Biology, 9,1–22.

Habel, J. C., Husemann, M., Finger, A., Danley, P. D., & Zachos, F. E. (2014). The relevance of time series in molecular ecology and conservation biology. Biological Reviews, 89(2), 484–492. https://doi.org/10.1111/brv.12068

Hagmeier, A. (1925). Vorläufiger Bericht über die vorbereitenden Untersuchungen der Bodenfauna der Deutschen Bucht mit dem Petersen-Bodengreifer. Ber. Dt. Wiss. Kommn Meeresforsch., 1.

Harrison, S. (1991). Local extinction in a metapopulation context: An empirical evaluation. Biological Journal of the Linnean Society, 42(1–2), 73–88.

Hayer, S., Bick, A., Brandt, A., Ewers-Saucedo, C., Fiege, D., Füting, S., Krause-Kyora, B., Michalik, P., Reinicke, G.-B., & Brandis, D. (2019). Coming and going – Historical distributions of the European oyster Ostrea edulis Linnaeus, 1758 and the introduced slipper limpet Crepidula fornicata Linnaeus, 1758 in the North Sea. PLOS ONE, 14(10), e0224249. https://doi.org/10.1371/journal.pone.0224249

Hayer, S., Brandis, D., Immel, A., Susat, J., Torres-Oliva, M., Ewers-Saucedo, C., & Krause-Kyora, B. (2021). Phylogeography in an "oyster" shell provides first insights into the genetic structure of an extinct Ostrea edulis population. Scientific Reports, 11(1), 1–10.

Homburger, J. R., Neben, C. L., Mishne, G., Zhou, A. Y., Kathiresan, S., & Khera, A. V. (2019).

Low coverage whole genome sequencing enables accurate assessment of common variants and calculation of genome-widepolygenic scores. Genome Medicine, 11,1-12.

Johannesson, K., Smolarz, K., Grahn, M., & André, C. (2011). The Future of Baltic Sea Populations: Local Extinction or Evolutionary Rescue? AMBIO, 40(2), 179-190. https://doi.org/10.1007/s13280-010-0129-x

Kearse, M., Moir, R., Wilson, A., Stones-Havas, S., Cheung, M., Sturrock, S., Buxton, S., Cooper, A., Markowitz, S., Duran, C., Thierer, T., Ashton, B., Mentjies, P., & Drummond, A. (2012). Geneious Basic: An integrated and extendable desktop software platform for the organization and analysis of sequence data (version 8.0.3). Bioinformatics, 28(12), 1647-1649.

Korneliussen, T. S., Albrechtsen, A., & Nielsen, R. (2014). ANGSD: Analysis of Next Generation Sequencing Data. BMC Bioinformatics, 15(1), 356. https://doi.org/10.1186/s12859-014-0356-4

Lapègue, S., Reisser, C., Harrang, E., Heurtebise, S., & Bierne, N. (2022). Genetic parallelism between European flat oyster populations at the edge of their natural range. Evolutionary Applications.

Launey, S., Ledu, C., Boudry, P., Bonhomme, F., & Naciri-Graven, Y. (2002). Geographic Structure in the European Flat Oyster (Ostrea edulis L.) as Revealed by Microsatellite Polymorphism. Journal of Heredity, 93(5), 331-351. https://doi.org/10.1093/jhered/93.5.331

Li, H. (2018). Minimap2: Pairwise alignment for nucleotide sequences. Bioinformatics, 34(18), 3094-3100.

Li, S., Yan, B., Li, T. K., Lu, J., Gu, Y., Tan, Y., Gong, F., Lam, T.-W., Xie, P., & Wang, Y. (2023). Ultra-low-coverage genome-wide association study—Insights into gestational age using 17,844 embryo samples with preimplantation genetic testing. Genome Medicine, 15(1), 10.

Lou, R. N., Jacobs, A., Wilder, A. P., & Therkildsen, N. O. (2021). A beginner's guide to low-coverage whole genome sequencing for population genomics. Molecular Ecology, 30(23), 5966-5993.

Luttikhuizen, P. C., Drent, J., & Baker, A. J. (2003). Disjunct distribution of highly diverged mitochondrial lineage clade and population subdivision in a marine bivalve with pelagic larval dispersal. Molecular Ecology, 12(8), 2215-2229. https://doi.org/10.1046/j.1365-294X.2003.01872.x

Maes, G., & Volckaert, F. (2002). Clinal genetic variation and isolation by distance in the

European eel Anguilla anguilla (L.). Biological Journal of the Linnean Society, 77(4), 509–521.

Maggs, C. A., Castilho, R., Foltz, D., Henzler, C., Jolly, M. T., Kelly, J., Olsen, J., Perez, K. E., Stam, W., Väinölä, R., Viard, F., & Wares, J. (2008). Evaluating signatures of glacial refugia for North Atlantic benthic marine taxa. Ecology, 89(sp11), S108–S122. https://doi.org/10.1890/08-0257.1

Manni, M., Berkeley, M. R., Seppey, M., & Zdobnov, E. M. (2021). BUSCO: assessing genomic data quality and beyond. Current Protocols, 1(12), e323.

Meisner, J., Albrechtsen, A., & Hanghøj, K. (2021). Detecting selection in low-coverage high-throughputsequencing data using principal component analysis. BMC Bioinformatics, 22(1), 1–13.

Meyerjürgens, J., Badewien, T. H., Garaba, S. P., Wolff, J.-O., & Zielinski, O. (2019). A state-of-the-artcompact surface drifter reveals pathways of floating marine litter in the German bight. Frontiers in Marine Science, 6,58.

Mi, H., Muruganujan, A., & Thomas, P. D. (2012). PANTHER in 2013: Modeling the evolution of gene function, and other gene attributes, in the context of phylogenetic trees. Nucleic Acids Research, 41(D1), D377–D386.

Möbius, K. A. (1870). Ueber Austern-undMiesmuschelzucht und die Hebung derselben an den norddeutschenKüsten.VerlagvonWiegandtundHempel. https://opacplus.bsb-muenchen.de/Vta2/bsb11012000/bsb:BV020325337

Möbius, K. A. (1877). Die Auster und die Austernwirthschaft.Parey.

Neudecker, T. (1979). Zur Qualität von Austern aus der Flensburger Förde. Informationen Für Die Fischwirtschaft, 26(5), 142–143.

Neudecker, T. (1990). Genutzte Muscheln und Schnecken (Exploited Bivalves and Snails). In Warnsignale aus der Nordsee (p. 431). Paul Parey.

Newman, D., & Pilson, D. (1997). Increased probability of extinction due to decreased genetic effective population size: Experimental populations of Clarkia pulchella. Evolution, 51(2), 354–362.

Orr, H. A., & Unckless, R. L. (2008). Population extinction and the genetics of adaptation. The American Naturalist, 172(2), 160–169.

Otto, L., Zimmerman, J., Furnes, G., Mork, M., Saetre, R., & Becker, G. (1990). Review of the physical oceanography of the North Sea. Netherlands Journal of Sea Research, 26(2–4), 161–238.

Paradis, E., Claude, J., & Strimmer, K. (2004). APE: analyses of phylogenetics and evolution in

Rlanguage.Bioinformatics, 20,289–290.
Peltzer, A., Jäger, G., Herbig, A., Seitz, A., Kniep, C., Krause, J., & Nieselt, K. (2016). EAGER: efficient ancient genome reconstruction. Genome Biology, 17(1), 1–14.
RCoreTeam.(2019).R: A Language and Environment for Statistical Computing[Computer software]. R Foundation for Statistical Programming. https://www.R-project.org/
Raxworthy, C. J., & Smith, B. T. (2021). Mining museums for historical DNA: advances and challenges in museomics. Trends in Ecology & Evolution.
Robinson, J. A., Räikkönen, J., Vucetich, L. M., Vucetich, J. A., Peterson, R. O., Lohmueller, K. E., & Wayne, R. K. (2019). Genomic signatures of extensive inbreeding in Isle Royale wolves, a population on the threshold of extinction. Science Advances, 5(5), eaau0757.
Roman, J. O. E., & Palumbi, S. R. (2004). A global invader at home: Population structure of the green crab, Carcinus maenas, in Europe. Molecular Ecology, 13(10), 2891–2898. https://doi.org/10.1111/j.1365-294X.2004.02255.x
Rosche, C., Baasch, A., Runge, K., Brade, P., Träger, S., Parisod, C., & Hensen, I. (2022). Tracking population genetic signatures of local extinction with herbarium specimens. Annals of Botany, 129(7), 857–868.
Rosner, B. (1975). On the detection of many outliers. Technometrics, 17(2), 221–227.
Saavedra, C., Zapata, C., & Alvarez, G. (1995). Geographical patterns of variability at allozyme loci in the European oyster Ostrea edulis. Marine Biology, 122(1), 95–104.
Skotte, L., Korneliussen, T. S., & Albrechtsen, A. (2013). Estimating individual admixture proportions from next generation sequencing data. Genetics, 195(3), 693–702.
Smit, A., Hubley, R., & Green, P. (2013). RepeatMasker Open-4.0[Computer software]. http://www.repeatmasker.org
Stanke, M., Keller, O., Gunduz, I., Hayes, A., Waack, S., & Morgenstern, B. (2006). AUGUSTUS: ab initio prediction of alternative transcripts. Nucleic Acids Res, 34. https://doi.org/10.1093/nar/gkl200
Stanke, M., & Morgenstern, B. (2005). AUGUSTUS: a web server for gene prediction in eukaryotes that allows user-defined constraints. Nucleic Acids Res, 33. https://doi.org/10.1093/nar/gki458
Tajima, F. (1989). Statistical method for testing the neutral mutation hypothesis by DNA polymorphism. Genetics, 123,595–595.
Tarnowska, K., Krakau, M., Jacobsen, S., Wołowicz, M., Féral, J.-P., & Chenuil, A. (2012). Comparative phylogeography of two sister (congeneric) species of cardiid bivalves: Strong influence of habitat, life history and post-glacial history. Estuarine, Coastal and

Shelf Science, 107,150-158.https://doi.org/10.1016/j.ecss.2012.05.007

Thomas, P. D., Ebert, D., Muruganujan, A., Mushayahama, T., Albou, L., & Mi, H. (2022). PANTHER: Making genome-scale phylogenetics accessible to all. Protein Science, 31(1), 8-22.

Toussaint, E. F., Gauthier, J., Bilat, J., Gillett, C. P., Gough, H. M., Lundkvist, H., Blanc, M., Muñoz-Ramírez, C. P., & Alvarez, N. (2021). HyRAD-X exome capture museomics unravels giant ground beetle evolution. Genome Biology and Evolution.

Turvey, S. T., Crees, J. J., Li, Z., Bielby, J., & Yuan, J. (2017). Long-termarchives reveal shifting extinction selectivity in China's postglacial mammal fauna. Proceedings of the Royal Society B: Biological Sciences, 284(1867), 20171979.

UniProt Consortium. (2021). UniProt: The universal protein knowledgebase in 2021. Nucleic Acids Research, 49(D1), D480-D489.

Vera, M., Carlsson, J., Carlsson, J. E., Cross, T., Lynch, S., Kamermans, P., Villalba, A., Culloty, S., & Martinez, P. (2016). Current genetic status, temporal stability and structure of the remnant wild European flat oyster populations: Conservation and restoring implications. Marine Biology, 163(12), 239. https://doi.org/10.1007/s00227-016-3012-x

Wan, X., Jiang, G., Yan, C., He, F., Wen, R., Gu, J., Li, X., Ma, J., Stenseth, N. C., & Zhang, Z. (2019). Historical records reveal the distinctive associations of human disturbance and extreme climate change with local extinction of mammals. Proceedings of the National Academy of Sciences, 116(38), 19001-19008.

Weisenfeld, N. I., Kumar, V., Shah, P., Church, D. M., & Jaffe, D. B. (2017). Direct determination of diploid genome sequences. Genome Research, 27(5), 757-767.

Wickham, H. (2011). The split-apply-combinestrategyfor data analysis. Journal of Statistical Software, 40(1), 1-29.

Yonge, C. M. (1960). Oysters (Vol. 18). Collins.

Zheng, G. X., Lau, B. T., Schnall-Levin, M., Jarosz, M., Bell, J. M., Hindson, C. M., Kyriazopoulou-Panagiotopoulou, S., Masquelier, D. A., Merrill, L., & Terry, J. M. (2016). Haplotyping germline and cancer genomes with high-throughputlinked-readsequencing. Nature Biotechnology, 34(3), 303-311.
